# The efficacy versus evidence quality of multicomponent exercise for cognitive health in older adults: an umbrella review

**DOI:** 10.3389/fnagi.2025.1719179

**Published:** 2025-12-15

**Authors:** Weibao Liang, Chuannan Liu, Xujie Yan, Shuting Xu, Jianmin Dai, Wenbai Huang

**Affiliations:** 1 Guangdong Provincial Key Laboratory of Speed Capability Research, Su Bingtian Center for Speed Research and Training, School of Physical Education, Jinan University, Guangzhou, China; 2 School of Physical Education and Health, Zunyi Medical University, Zunyi, China; 3 School of Physical Education and Sports Science, Hengyang Normal University, Hengyang, China; 4 The First Affiliated Hospital of Jinan University, Guangzhou, China; 5 College of Sports Science, Kyungnam University, Changwon, Republic of Korea

**Keywords:** multicomponent exercise, cognitive function, older adults, physical activity, executive function

## Abstract

**Introduction:**

Multicomponent exercise (MCE) is a promising strategy for enhancing cognitive function in older adults. This umbrella review aimed to synthesize and critically appraise the evidence from systematic reviews and meta-analyses on the effects of multicomponent physical exercise on cognition in this population.

**Methods:**

An umbrella review of systematic reviews with or without meta-analysis was conducted. Six electronic databases (PubMed, Web of Science, Embase, Scopus, SPORTDiscus, and the Cochrane Library) were searched from their inception to September 2025 to identify eligible studies. The methodological quality of the included reviews was assessed using the AMSTAR-2 tool, and the overall certainty of the evidence for key outcomes was evaluated using the GRADE framework.

**Results:**

The synthesis included 27 systematic reviews. MCE demonstrated consistently statistically significant, moderate positive effects on global cognitive function (SMD = 0.45) and executive function (SMD = 0.31). regarding memory, while the overall effect and verbal memory showed significant improvements, specific sub-domains such as working memory and delayed memory did not reach statistical significance. Similarly, no significant effect was observed for attention/processing speed. Despite these positive findings, the methodological quality of the majority of the included reviews (22 of 27) was rated as “Low” or “Critically Low” by AMSTAR-2. Consequently, the certainty of the evidence according to GRADE was predominantly “Low” to “Very Low” for most cognitive outcomes, with “Moderate” certainty achieved only for global cognitive function.

**Conclusion:**

Multicomponent exercise is an effective intervention for improving global cognitive function and executive function in older adults. While benefits for specific memory domains and processing speed were less consistent, these findings support the clinical and public health promotion of MCE, while simultaneously highlighting an urgent need for more methodologically rigorous research to solidify the evidence base.

**Systematic review registration:**

https://www.crd.york.ac.uk/PROSPERO/view/ CRD420251161230, identifier CRD420251161230.

## Graphical Abstract

**Figure:**
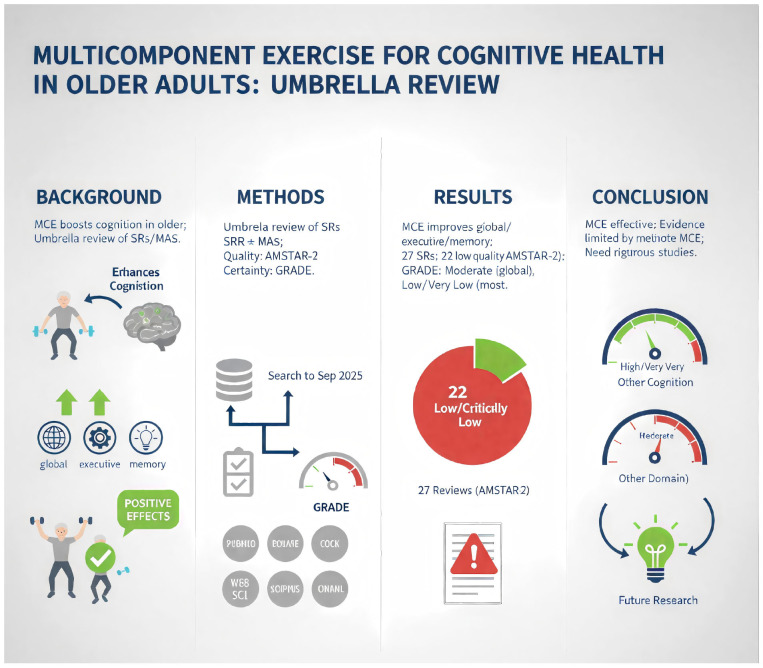
The impact of multicomponent exercise on cognitive function in older adults. This umbrella review synthesized evidence from 27 systematic reviews and meta-analyses. The findings indicate that multicomponent exercise, which combines aerobic, resistance, and balance training, significantly improves multiple cognitive domains, including global cognitive function, executive function, and memory. However, the methodological quality of the existing evidence base is predominantly low to moderate, highlighting a need for more rigorous future research to solidify these promising findings.

## 1 Introduction

The world is undergoing an unprecedented demographic shift. The proportion of adults aged 60 and over is increasing at a faster rate than any other age group (Bloom et al., 2015; Tu et al., 2022). This global aging phenomenon presents profound implications for public health, social care, and economic stability. Among the most pressing challenges associated with an aging population is the rising prevalence of age-related cognitive decline (Bishop et al., 2010; Yang et al., 2023). This decline ranges from subtle memory lapses to severe neurodegenerative conditions, such as Alzheimer’s disease and other dementias (Gonzales et al., 2022). Cognitive impairment significantly compromises an individual’s quality of life (Hussenoeder et al., 2020). Furthermore, it increases the burden on families and caregivers while placing immense strain on global healthcare systems (Gauthier et al., 2006; Whitehouse and Moody, 2006; Tahami Monfared et al., 2022).

The trajectory of cognitive aging exists on a continuum. In research and clinical settings, cognitive health is often evaluated through “global cognitive function,” which refers to the overall functional integrity of the brain’s cognitive processes. It represents a generalized construct that summarizes performance across multiple specific domains, including episodic memory, executive function, attention, language, and visuospatial skills (Lezak et al., 2012; Sachdev et al., 2014). Typically, this is quantified using composite scores from extensive neuropsychological batteries (e.g., ADAS-Cog) (Rosen et al., 1984) or standardized screening instruments such as the Mini-Mental State Examination (MMSE) (Folstein et al., 1975) and the Montreal Cognitive Assessment (MoCA) (Nasreddine et al., 2005). While some cognitive decline is normal with aging, many older adults experience Mild Cognitive Impairment (MCI). This transitional state involves objective deficits that do not yet meet dementia criteria but significantly increase the risk of progression to severe neurodegeneration (Petersen et al., 2014). Given the continued lack of effective pharmacological treatments, identifying non-pharmacological strategies is urgent (Whitty, 2020; Couch, 2023).

Over the past two decades, physical exercise has emerged as a cornerstone of healthy aging. A robust body of evidence indicates that regular physical activity can enhance cognitive function, preserve brain structure, and potentially reduce the risk of dementia (Kramer and Erickson, 2007; Mandolesi et al., 2018; Erickson et al., 2019). While various exercise modalities, such as aerobic and resistance training, have been studied independently for their cognitive benefits, there is increasing interest in integrated approaches that better reflect the complex physical demands of daily life.

Multicomponent exercise (MCE) has become a particularly promising intervention for older adults. In the context of this review, MCE refers to a structured physical exercise program that incorporates elements from at least two of the following training modalities: aerobic exercise (e.g., walking, jogging), resistance training (e.g., resistance bands, dumbbells), balance training (e.g., single-leg stance, gait training), flexibility training (e.g., stretching), as well as coordination and agility training (Bird et al., 2011; Li et al., 2021). By challenging the cardiovascular, musculoskeletal, and neuromuscular systems concurrently, MCE may induce more comprehensive and synergistic benefits than any single modality alone (Mandolesi et al., 2018; Labata-Lezaun et al., 2023). Furthermore, the inherent variety and complexity of MCE often require greater cognitive engagement—such as learning new movement patterns and maintaining attention—which may provide an additive stimulus for enhancing brain function (de Asteasu et al., 2017; Basak et al., 2020). Crucially, this cognitive demand shares mechanistic similarities with intellectual activities like Chinese Chess and Calligraphy, which rely heavily on pattern recognition and visuo-spatial navigation. Engaging in such complex tasks is thought to bolster specific cognitive domains, including short-term working memory and delayed memory recall. Recent integrative functional and structural neuroimaging evidence supports this view, indicating that continuous complex learning—analogous to the neuroplasticity observed during periods of higher education—can lead to quantitative brain adaptations that foster individual long-term wisdom, creativity, and potential neuroprotection (Zhou, 2023).

Reflecting this growing interest, a rapid proliferation of systematic reviews and meta-analyses has sought to synthesize the evidence on MCE and cognition (Basak et al., 2020; Borges-Machado et al., 2021; Santos Lopes da Silva et al., 2023). Currently, a comprehensive synthesis that critically appraises and integrates these findings is lacking. This umbrella review aims to bridge this gap, providing clinicians and policymakers with a rigorous evaluation of the evidence to guide decision-making.

## 2 Materials and methods

### 2.1 Protocol and reporting

This umbrella review was conducted and reported in accordance with the Preferred Reporting Items for Systematic Reviews and Meta-Analyses (PRISMA) 2020 statement (Page et al., 2021). The review protocol was established a priori to define the research questions, search strategy, and inclusion criteria. The protocol for this study was pre-registered on the International Prospective Register of Systematic Reviews (PROSPERO), under the registration number: CRD420251161230.

### 2.2 Search strategy

A systematic and comprehensive literature search was conducted in six electronic databases: PubMed, Web of Science, Embase, Scopus, SPORTDiscus, and the Cochrane Library. The inclusion of SPORTDiscus was intended to capture exercise-specific journals and literature that may be omitted by general medical databases. The search was performed from the inception of each database to September 2025. The search strategy combined keywords and subject headings related to three core concepts: (1) the population (e.g., “older adults,” “elderly,” “aging”); (2) the intervention (e.g., “multicomponent exercise,” “combined training,” “mixed exercise”); and (3) the study design (e.g., “systematic review,” “meta-analysis”). Detailed search strategy for each database is available in Supplementary material. For instance, the specific search string used for PubMed was: (“older adults” OR “elderly” OR “aged”). AND (“multicomponent exercise”. OR “combined training.” OR “concurrent training”). AND (“systematic review” OR “meta-analysis”). Additionally, the reference lists of included reviews and relevant publications were manually screened to identify any potentially eligible studies.

### 2.3 Inclusion and exclusion criteria

Studies were included in this umbrella review if they met the following criteria:

- Population: Older adults, defined as having a mean or median age of 60 years or older. Reviews focusing on specific cognitive statuses [e.g., cognitively healthy, Mild Cognitive Impairment (MCI), frailty] were all eligible.
- Intervention: Multicomponent exercise (MCE), defined as a structured exercise program incorporating elements from at least two of the following modalities: aerobic, resistance/strength, balance, and flexibility training.
- Comparator: Any non-MCE control group. This included inactive controls (e.g., no intervention, usual care) or active controls (e.g., single-intervention exercises like walking only). The inclusion of active controls (SIE) was intended to allow for the evaluation of the specific “added value” or synergistic effects of the multicomponent approach compared to single-intervention exercises.
- Outcomes: The review must have reported on at least one standardized measure of cognitive function. This included Global Cognitive Function (typically assessed by screening tools such as the MMSE or MoCA), Executive Function, Memory (including subtypes such as working, immediate, delayed, and verbal memory), Attention, or Processing Speed.
- Study Design: Published systematic reviews with or without a quantitative meta-analysis.

Exclusion criteria included original research articles (e.g., individual RCTs), narrative reviews without a systematic search methodology and conference abstracts.

### 2.4 Study selection and data extraction

All records retrieved from the database search were imported into a reference management software, and duplicates were removed. Two reviewers independently screened the titles and abstracts of the remaining records against the eligibility criteria. The full texts of potentially relevant articles were then retrieved and assessed for final inclusion by the same two reviewers. Any disagreements at either stage of the screening process were resolved through discussion or, if necessary, by consulting a third reviewer.

A standardized data extraction form was developed and used by two independent reviewers to extract relevant information from the finally included studies. Discrepancies in extracted data were resolved by consensus. The extracted data included: (1) general characteristics (author, year of publication); (2) study design details (number and type of primary studies); (3) population characteristics (sample size, age, sex, cognitive status); (4) intervention and comparator details; (5) cognitive outcome measures; (6) the results of any quantitative meta-analyses (pooled effect sizes, 95% confidence intervals, heterogeneity statistics); and (7) the authors’ conclusions regarding the methodological quality of their included primary studies.

### 2.5 Methodological quality assessment

The methodological quality of each included systematic review was independently assessed by two reviewers using the AMSTAR-2 (A MeaSurement Tool to Assess systematic Reviews 2) tool. The AMSTAR-2 is a 16-item instrument that evaluates the rigor of the systematic review process, with seven items designated as critical domains. According to the AMSTAR-2 guidance (Shea et al., 2017), the overall confidence in the results was rated as: “High” (zero or one non-critical weakness), “Moderate” (more than one non-critical weakness), “Low” (one critical weakness with or without non-critical weaknesses), or “Critically Low” (more than one critical weakness). Any disagreements in the quality ratings were resolved by consensus.

### 2.6 Data Synthesis and certainty of evidence

A narrative synthesis was conducted to summarize the characteristics, key findings, and methodological quality of the included reviews. For the quantitative synthesis, we specifically extracted the pooled effect sizes [Standardized Mean Differences (SMD) or Mean Differences (MD)] and their 95% confidence intervals as reported by the original meta-analyses. Systematic reviews that did not provide quantitative pooled estimates were excluded from the generated forest plots and were summarized narratively.

Quantitative results from the meta-analyses were extracted and tabulated, organized by specific cognitive outcome domains. Regarding publication bias, we did not perform a de novo funnel plot or Egger’s test on the set of included systematic reviews, given the aggregated nature of the data. Instead, we extracted the publication bias findings (e.g., results from Egger’s tests or funnel plot inspections) reported within each included review. These reported assessments were used to evaluate the “Publication Bias” domain in the subsequent GRADE assessment. Effect sizes were extracted as Standardized Mean Differences (SMD), predominantly Hedges’ g (to correct for small sample sizes) or Cohen’s d, as reported by the primary reviews.

The overall certainty of the evidence for the main cognitive outcomes was assessed using the GRADE (Grading of Recommendations, Assessment, Development and Evaluation) framework. For each outcome, the evidence from the included meta-analyses of RCTs started at “High” certainty. The rating was then downgraded by one level for “serious” limitations or by two levels for “very serious” limitations across five domains: risk of bias (informed by our AMSTAR-2 assessment and the reviews’ own quality assessments), inconsistency (unexplained heterogeneity, informed by the *I*^2^ statistic), indirectness (concerns regarding the generalizability or applicability of the evidence to the research question), imprecision (small number of studies or wide confidence intervals), and publication bias. Consequently, the final certainty of evidence was classified into one of four levels: “High” (very confident in the effect estimate), “Moderate” (moderately confident), “Low” (limited confidence), or “Very Low” (very little confidence).

## 3 Results

### 3.1 Study selection

**FIGURE 1:**
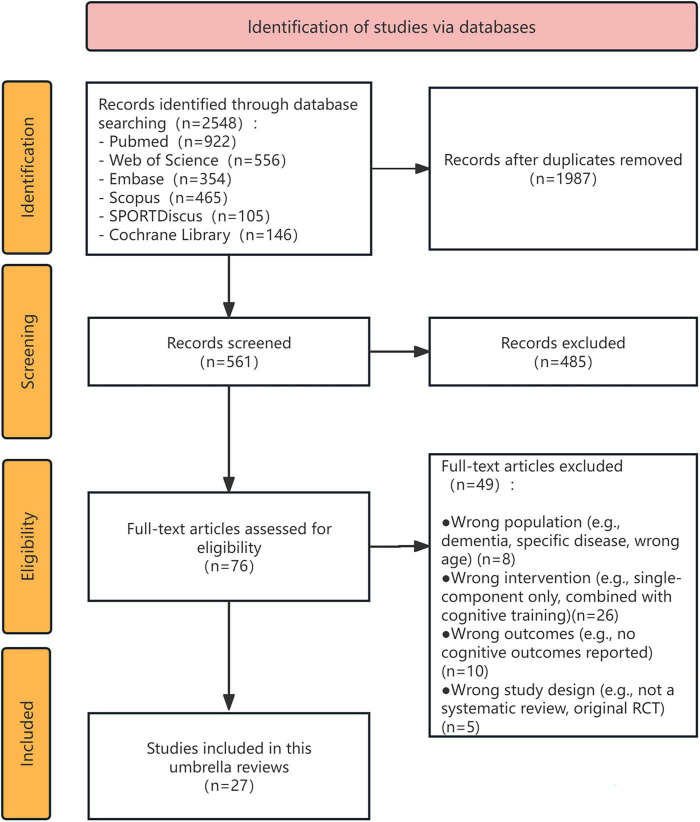
PRISMA flow diagram of the study selection process. The diagram illustrates the flow of information through the different phases of the umbrella review. It details the number of records identified through database searching, the number of duplicates removed, the number of records screened, the number of full-text articles assessed for eligibility, and the final number of systematic reviews included in the synthesis, with reasons for exclusion at each stage.

The study selection process is illustrated in the PRISMA flow diagram (Figure 1). The initial search across six electronic databases (PubMed, Web of Science, Embase, Scopus, SPORTDiscus, and the Cochrane Library) yielded a total of 2,548 records. After duplicates were removed, 1,987 unique articles remained for screening. During the title and abstract screening phase, 485 records were excluded, leaving 76 full-text articles to be assessed for eligibility. Upon full-text review, a further 49 studies were excluded. Ultimately, 27 systematic reviews (Tseng et al., 2011; Carvalho et al., 2014; de Asteasu et al., 2017; Northey et al., 2017; Falck et al., 2019; Sanders et al., 2019; Biazus-Sehn et al., 2020; Bliss et al., 2020; Wang et al., 2020, 2024; Xiong et al., 2020; Cai et al., 2021; Ahn and Kim, 2022; Gallardo-Gómez et al., 2022; Li et al., 2022; Mello et al., 2022; da Silva et al., 2022, 2023; Xu et al., 2023; Alowaydhah et al., 2024; Cerda-Vega et al., 2024; Luo et al., 2024; Ni et al., 2024; Sirikul et al., 2024; Jia et al., 2025; Liu et al., 2025; Vafa et al., 2025) met the full inclusion criteria and were included in the final synthesis for this umbrella review.

### 3.2 Characteristics of included reviews

The detailed characteristics of the 27 included reviews are summarized in Table 1. The publication dates ranged from 2011 to 2025, providing a comprehensive overview of the evidence. The scope of these reviews was extensive, collectively synthesizing data from hundreds of primary randomized controlled trials and encompassing tens of thousands of participants.

**TABLE 1:** Characteristics of the included studies.

Study	No. of studies and type	Total sample size (N)	Population characteristics	Population cognitive status	Intervention details	Comparator	Outcomes	Research question/objective
Ahn and Kim (2022)	22 RCTs	1,916	Age ≥ 60 years	Mild cognitive impairment (MCI)	Subgroup analysis including aerobic, resistance, multicomponent, and neuromotor exercise.	No intervention, usual care, health education, stretching.	Global cognitive function	To identify the effects of different exercise modalities on global cognitive function in older adults with MCI and to determine the optimal methods.
Alowaydhah et al. (2024)	57 controlled studies (38 in meta-analysis)	6,152 (Healthy: 2,370; Frail: 3,782)	Age ≥ 65 years; Healthy mean 74.6y, Frail mean 80.3y.	Healthy older adults; frail older adults	Combination of multiple elements from strength, flexibility, balance, coordination, and aerobic exercise.	Non-exercise control group	Cognitive function, physical function, psychological health	To determine if exercise prescription needs to be tailored for frail compared to healthy older adults.
de Asteasu et al. (2017)	21 RCTs	>1,400 (260 in MCE groups)	Age > 65 years	Healthy older adults without known cognitive impairment	Combination of aerobic, resistance, and other training modalities (e.g., balance, flexibility).	Stretching/toning, no-exercise active control, no intervention.	Cognitive function (executive function, memory)	To analyze the effects of different exercise modalities on cognition in healthy older adults.
Biazus-Sehn et al. (2020)	32 RCTs	2,932	Mean age ≥ 60 years, 63.8% female.	Mild cognitive impairment (MCI)	Subgroup analysis for multicomponent exercise.	Non-exercise control groups (e.g., usual care, social activities).	Global cognition, executive function, delayed recall, verbal fluency, attention/processing speed	To evaluate the effects of physical exercise on the cognitive function of older adults with MCI.
Bliss et al. (2020)	Not specified	Not specified	Age > 50 years	Healthy, MCI, post-stroke, Alzheimer’s, etc.	Combination of at least two exercise forms, e.g., aerobic ++ resistance.	Active or passive control groups.	Cerebrovascular function, cognition, neuroanatomical adaptations	To examine the benefits of exercise training on cerebrovascular and cognitive function in aging.
Cai et al. (2021)	28 RCTs	2,063	Age range 62–86 years	Normal cognition (17 studies); MCI (11 studies)	Multicomponent exercise (MCE) as one of the analyzed types.	Active control (stretching, education) and passive control (no intervention).	Working memory	To investigate the effects of physical exercise on the working memory of older adults and to identify moderators.
Carvalho et al. (2014)	19 longitudinal or experimental studies	13,874	Age > 60 years	Healthy and with cognitive impairment	Included “multiple intervention” or “combined training” categories.	Control groups without intervention.	Cognitive function	To verify the effects of physical activity on the cognitive functions of individuals over 60 years of age.
Cerda-Vega et al. (2024)	11 experimental longitudinal studies	636	Age > 60 years	Cognitively healthy older adults	Included aerobic, strength, and “combined exercise” (i.e., multicomponent).	Control group without physical exercise.	Attention, memory, processing speed, executive function, etc.	To synthesize appropriate exercise parameters (intensity, duration, frequency, type) for different cognitive variables.
Falck et al. (2019)	48 RCTs	6,281	Age ≥ 55 years	Cognitively normal, MCI, and dementia (stratified reporting)	Subgroup analysis for “mixed training” (i.e., multicomponent).	Non-active control (usual care, wait-list, education).	Cognitive function (global, executive function, memory, processing speed)	To evaluate the impact of exercise training on cognitive function among older adults.
Gallardo-Gómez et al. (2022)	79 RCTs	8,114	Mean age > 60 years	Healthy, MCI, or dementia (subgroup analysis)	Analyzed as an independent intervention type in a network meta-analysis.	No intervention, usual care, or active control.	Cognitive function	To determine the optimal dose and type of exercise to improve cognitive function in older adults.
Jia et al. (2025)	43 RCTs	3,924	Mean age > 60 years	Mild cognitive impairment (MCI)	Analyzed as an independent intervention type in a network meta-analysis.	Usual care, health education, etc.	Global cognition, executive function	To compare the relative efficacy of different exercise interventions on cognitive function in older adults with MCI.
Li et al. (2022)	24 RCTs	2,130	Age > 60 years	Mild cognitive impairment (MCI)	Subgroup analysis for “multicomponent training”.	Non-exercise intervention	Global cognitive function, executive function	To assess the effects of exercise training on cognitive function in adults with MCI.
Liu et al. (2025)	15 RCTs	1,208	Mean age 75.8 years	Mild cognitive impairment (MCI)	Specifically focused on multicomponent exercise.	Health education, usual care, social activities.	Global cognitive function, executive function, memory	To evaluate the effects of multi-component exercise on cognitive function in older adults with MCI.
Luo et al. (2024)	10 RCTs	895	Mean age > 65 years, 73% female.	Cognitive Frailty	Specifically focused on multicomponent exercise.	No intervention, usual care, health education.	Global cognition, executive function, memory	To evaluate the effectiveness of multicomponent exercise in older adults with cognitive frailty.
Mello et al. (2022)	18 RCTs	3,365	Mean age > 60 years	Frail older adults	Specifically focused on multicomponent exercise training.	Usual care, health education, etc.	Intrinsic capacity (including cognitive domain)	To assess the effects of multicomponent exercise training on the intrinsic capacity of frail older adults.
Ni et al. (2024)	27 RCTs	3,618	Mean age 72.8 years	Cognitively healthy; cognitively impaired (subgroup analysis)	Intervention was “complex exercise,” defined as including at least two training modalities.	Active or passive control groups	Cognitive performance (global cognition, executive function, memory, etc.)	To examine the effect of complex exercise interventions on cognitive performance in older adults.
Northey et al. (2017)	36 studies	2,871	Age > 50 years	With or without cognitive impairment	Subgroup analysis included “combined training” (aerobic + resistance).	Control conditions (e.g., usual care).	Attention, processing speed, executive function, memory	To evaluate exercise interventions for cognitive function in adults older than 50.
Sanders et al. (2019)	62 RCTs	6,438	Mean age 69.3 years	With or without cognitive impairment	Analysis included “combined (aerobic + resistance) training”.	Active or passive control groups.	Cognitive function	To explore the dose-response relationship between exercise and cognitive function.
da Silva et al. (2022)	42 RCTs	4,286	Mean age ≥ 60 years, 69% female.	Healthy or with clinical conditions	Specifically analyzed “multicomponent exercise programs”.	No or minimal intervention.	Mental health and cognitive function	To assess the effects of chronic physical exercise or multicomponent exercise on the mental health and cognitive function of older adults.
da Silva et al. (2023)	22 RCTs	2,746	Mean age ≥ 60 years, 72.8% female.	Older adults without cognitive impairment	Specifically focused on multicomponent training.	No intervention or active control.	Global cognition, executive function, memory, attention, processing speed	To determine if multicomponent training improves cognitive function in older adults without cognitive impairment.
Sirikul et al. (2024)	22 RCTs	2,492	Age ≥ 60 years	Frail or pre-frail older adults	Compared four interventions, including “multicomponent exercise only”.	Usual care or no intervention.	Physical frailty and cognitive function	To assess the impact of multicomponent exercise and/or nutritional supplements on frailty and cognitive function.
Tseng et al. (2011)	23 RCTs	2,906	Mean age 65–85 years	Healthy or with cognitive impairment	Included “multi-modal exercise” in its classification.	No intervention, social activities, etc.	Cognitive function	To assess the effectiveness of exercise on improving cognitive function in older people.
Vafa et al. (2025)	42 RCTs	4,204	Mean age 70.8 years, 71% female.	Healthy older adults	Subgroup analysis included “multicomponent” exercise.	Active or passive control	Cognitive functioning (global, executive function, memory)	To evaluate the effectiveness of real-life cognitive and physical interventions on cognitive functioning in healthy older adults.
Wang et al. (2020)	10 RCTs	973	Mean age 80.3 years, 65% female.	Frail, community-dwelling older adults	Specifically focused on multicomponent exercise.	Usual care, health education, social activities.	Neurocognition (global, executive function), BDNF	To evaluate the neurocognitive and BDNF changes from multicomponent exercise in frail older adults.
Wang et al. (2024)	17 RCTs	1,444	Age ≥ 60 years, 64.4% female.	Persons with Mild Cognitive Impairment (MCI)	Specifically focused on multicomponent exercise.	No intervention or active control (e.g., health education).	Cognitive function (global, executive function, etc.)	To evaluate the effects of multicomponent exercise on cognitive function in persons with MCI.
Xiong et al. (2020)	24 RCTs	2,933	Mean age ≥ 60 years	Cognitively healthy adults	Subgroup analysis included “mixed exercise” (i.e., multicomponent).	No intervention or active control.	Executive function	To assess the effects of physical exercise on executive function in cognitively healthy older adults.
Xu et al. (2023)	62 RCTs	7,748	Age > 50 years	Healthy or MCI	Subgroup analysis included “multicomponent training”.	No intervention or active control.	Cognitive function (global, executive function, memory)	To assess the effects of exercise for cognitive function in older adults.

The table provides a detailed summary of the 27 systematic reviews included in this umbrella review. For each review, the table outlines the number and type of primary studies, total participant sample size, key population characteristics, the cognitive status of the participants, a description of the intervention details focusing on multicomponent exercise, the types of comparators used, the main cognitive outcomes assessed, and the primary research question or objective of the review. MCE, Multicomponent Exercise; MCI, Mild Cognitive Impairment; RCTs, Randomized Controlled Trials; UC, Usual Care.

The target populations were consistently older adults. A key source of heterogeneity was the baseline cognitive status, with reviews focusing on cognitively healthy older adults, individuals with MCI, frail or cognitively frail older adults, or mixed populations.

The reviews focused on MCE interventions, typically compared against non-exercise controls such as usual care, health education, or social activities. Global cognitive function was the primary outcome reported, with frequent analyses of specific domains including executive function, memory, attention, and processing speed.

The quantitative findings from the included systematic reviews consistently demonstrated a beneficial effect of multicomponent exercise on cognitive function in older adults (Table 2). The most frequently assessed outcome was global cognitive function. Across numerous reviews, multicomponent exercise was found to have a statistically significant, small-to-moderate positive effect, with Standardized Mean Differences (SMDs) generally ranging from 0.24 to 0.65. One network meta-analysis reported a particularly large effect size (SMD = 1.52). Positive effects were also consistently reported for other key cognitive domains. Several meta-analyses showed significant improvements in executive function (SMDs ranging from 0.21 to 0.76) and various aspects of memory, including working memory (SMD = 0.38) and overall memory function (SMD = 0.21). While the direction of the effect was consistently positive, many of the pooled analyses reported moderate to substantial statistical heterogeneity (*I*^2^ > 50%), suggesting considerable variability in the results of the underlying primary studies.

**TABLE 2:** Summary of meta-analysis findings on the effects of multicomponent exercise on different cognitive domains in older adults.

Study	Cognitive outcome	No. of studies	Effect size [95% CI]	*I*^2^, *p*-value
Ahn and Kim (2022)	Global cognitive function	21	SMD = 0.65 [0.39, 0.91]	*I*^2^= 85%, *p*< 0.00001
Alowaydhah et al. (2024)	Cognitive function	8	SMD = 0.29 [0.10, 0.49]	*I*^2^ = 0%, *p* = 0.53
Biazus-Sehn et al. (2020)	Global cognitive function	18	SMD = 0.35 [0.17, 0.53]	*I*^2^ = 84%, *p*< 0.001
	Executive function	19	SMD = 0.21 [0.03, 0.40]	*I*^2^ = 68%, *p*<0.001
Cai et al. (2021)	Working memory	13	SMD = 0.38 [0.20, 0.56]	*I*^2^ = 0%, *p* = 0.61
Falck et al. (2019)	Global cognitive function	47	SMD = 0.24 [0.15, 0.33]	*I*^2^ = 65.87%, *p*< 0.001
Jia et al. (2025)	Global cognitive function	28	SMD = 1.52 [0.16, 2.87]	Inconsistency *p* = 0.745
Li et al. (2022)	Executive function	3	SMD = 0.44 [0.05, 0.83]	*I*^2^ = 0%, *p* = 0.40
Liu et al. (2025)	Global cognitive function	13	SMD = 0.31 [0.08, 0.55]	*I*^2^ = 82%, *p* < 0.00001
	Executive functions	4	SMD = 0.12 [-0.00, 0.24]	*I*^2^ = 0%, *p* = 0.46
Luo et al. (2024)	Global cognitive function	6	MD = 2.52 [1.05, 3.99]	*I*^2^ = 59%, *p* = 0.03
Ni et al. (2024)	Global cognitive function	27	SMD = 0.56 [0.34, 0.78]	*I*^2^ = 64%, *p*<0.05
	Executive function	13	SMD = 0.76 [0.41, 1.11]	Not Reported
	Cognitive inhibition	4	SMD = 0.79 [0.17, 1.41]	Not Reported
	Cognitive speed	5	SMD = 0.48 [0.13, 0.84]	Not Reported
	Memory	5	SMD = 0.07 [-0.20, 0.33]	Not Reported
Sanders et al. (2019)	Global cognitive function	13	SMD = 0.37 [CI Not Reported]	Not reported
	Executive function (healthy group)	23	SMD = 0.27 [CI Not Reported]	Not reported
	Memory (healthy group)	23	SMD = 0.24 [CI Not Reported]	Not reported
da Silva et al. (2023)	Global cognitive function	6	SMD = 0.58 [0.34, 0.81]	*I*^2^ = 11%, *p* = 0.35
	Attention/processing speed (TMT-A)	4	MD = -6.70 [-10.19, -3.21]	*I*^2^ = 51%, *p* = 0.11
Sirikul et al. (2024)	Global cognitive function	3	SMD = 0.33 [0.09, 0.57]	*I*^2^ = 0%, *p* = 0.45
Vafa et al. (2025)	Global cognitive function	7	SMD = 0.44 [0.12, 0.76]	*I*^2^ = 66.8%, *p* = 0.005
	Memory	7	SMD = 0.21 [0.02, 0.39]	*I*^2^ = 0%, *p* = 0.96
Wang et al. (2020)	Global cognitive function	10	WMD = 0.18 [0.02, 0.34]	*I*^2^ = 74%, *p*<0.001
Wang et al. (2024)	Global cognitive function	16	SMD = 0.44 [0.24, 0.64]	*I*^2^ = 69%, *p*<0.001
Xiong et al. (2020)	Executive function	10	SMD = 0.36 [0.11, 0.61]	*I*^2^ = 42%, *p* = 0.08
Xu et al. (2023)	Cognitive function	17	SMD = 0.35 [0.18, 0.52]	*I*^2^ = 65%, *p*<0.01

The table presents the pooled effect sizes extracted from the meta-analyses within the included systematic reviews, categorized by cognitive outcome. For each finding, the table specifies the source review, the number of primary studies included in that specific meta-analysis, the calculated pooled effect size with its 95% confidence interval, and the statistical heterogeneity. CI, Confidence Interval; *I*^2^, I-squared statistic for heterogeneity; MD, Mean Difference; SMD, Standardized Mean Difference; WMD, Weighted Mean Difference.

### 3.3 Methodological quality assessment of primary studies

The included systematic reviews utilized a variety of established tools to assess the methodological quality and risk of bias of their primary studies (Table 3). The most frequently employed instruments were the Physiotherapy Evidence Database (PEDro) scale and various versions of the Cochrane Risk of Bias (RoB) tool. Other tools such as the Jadad scale and the Agency for Healthcare Research and Quality (AHRQ) guidelines were also used by some reviews. Three reviews were narrative or descriptive and did not report a formal quality assessment of primary studies.

**TABLE 3:** Summary of methodological quality assessment tools and conclusions.

Author, year	Methodological quality assessment Tool used	Authors’ conclusion on methodological quality
Ahn and Kim (2022)	Cochrane’s risk of bias tool 2.0 (RoB 2.0)	Of the 22 included RCTs, 11 (50%) were “low risk,” 11 (50%) were of “some concerns,” and none were “high risk.”
Alowaydhah et al. (2024)	Physiotherapy evidence database (PEDro) scale	The methodological quality of the 57 included studies ranged from low to high, with a mean PEDro score of 5.5 (out of 10).
Biazus-Sehn et al. (2020)	Cochrane collaboration’s tool for assessing the risk of bias	Seven studies were considered to have a low risk of bias, while the remaining 20 studies presented a high risk of bias.
Bliss et al. (2020)	Not Reported (narrative review, no formal assessment conducted)	Not reported.
Cai et al. (2021)	Physiotherapy evidence database (PEDro) scale	The overall methodological quality was “fair to excellent,” with 15 studies rated as good (PEDro score ≥ 6), 7 as fair (4–5), and 6 as excellent (9–10).
Carvalho et al. (2014)	Agency for healthcare research and quality (AHRQ) methods reference guide	The majority of studies (15/27) were of “fair” quality, while three were “poor”; the overall risk of bias was “moderate” for most studies (16/27).
Cerda-Vega et al. (2024)	Physiotherapy evidence database (PEDro) scale	The included studies were rated as having “moderate to high” methodological quality.
da Silva et al. (2022)	Jadad scale	Most of the included studies were considered to be of “good quality.”
de Asteasu et al. (2017)	Cochrane handbook (section 8)	Methodological quality varied, with strengths in reporting follow-up but weaknesses in allocation concealment and blinding.
Falck et al. (2019)	Cochrane collaboration guidelines	The majority of studies that included participants with mixed cognitive or physical status were rated as “low quality.”
Gallardo-Gómez et al. (2022)	Cochrane risk of bias tool (RoB)	Sixteen studies had a low risk of bias, eighteen had some risk, and ten had a high risk; the overall quality of evidence was rated as “moderate” by GRADE.
Jia et al. (2025)	Cochrane risk of bias tool	Overall, 4 studies were assessed as “high risk,” and 29 studies were considered to have “moderate risk.”
Li et al. (2022)	Cochrane risk of bias tool	The quality of the included trials varied; about half did not state randomization or blinding methods, and allocation concealment was poorly reported in almost all trials.
Liu et al. (2025)	Cochrane collaboration’s tool for assessing risk of bias	Of the 13 included studies, 8 were assigned a level A quality rating, and 5 were assigned a level B rating.
Luo et al. (2024)	Cochrane risk of bias tool (for RCTs) and ROBINS-I tool (for non-RCTs)	Primary risk sources in RCTs were selection, performance, and detection bias. In non-RCTs, risks were from confounding and deviations from interventions.
Mello et al. (2022)	Not reported (descriptive review)	Not reported.
Ni et al. (2024)	Cochrane risk of bias (RoB) tool	High-risk literature was mainly at risk of selection bias, reporting bias, and other biases.
Northey et al. (2017)	Physiotherapy evidence database (PEDro) scale	The mean PEDro score for the 39 included articles was 6.7 out of 10 (range 5–9), which is generally considered “good” quality.
Sanders et al. (2019)	Physiotherapy evidence Database (PEDro) scale	The mean PEDro score was 6.2 (range: 4–8), which indicates a “moderate” methodological quality.
da Silva et al. (2023)	Cochrane risk of bias tool (RoB 2) and Physiotherapy evidence database (PEDro) scale	The methodological quality of the studies was classified as “good quality.”
Sirikul et al. (2024)	Physiotherapy evidence database (PEDro) scale	The methodological quality of the included studies was “fair to good” (PEDro score > 4/10).
Tseng et al. (2011)	Jadad scale	This review included 12 “medium to high-quality” randomized controlled trials.
Vafa et al. (2025)	Physiotherapy evidence database (PEDro) scale	The average methodological quality score was 6.75 out of 10, indicating “good quality.”
Wang et al. (2020)	Physiotherapy evidence database (PEDro) scale	The mean PEDro score was 5.9 (range, 4–7), indicating “fair” methodological quality.
Wang et al. (2024)	Cochrane risk-of-bias 2 (RoB 2) tool	The overall risk of bias was low in five studies, while 11 studies showed “some concerns.”
Xiong et al. (2020)	Physiotherapy evidence database (PEDro) scale	The methodological quality was “moderate to high,” with an average PEDro score of 6.2.
Xu et al. (2023)	Physiotherapy evidence database (PEDro) scale	The mean PEDro score was 6.24, indicating a “moderate” methodological quality.

The table lists the specific methodological quality or risk of bias assessment tools used by each included systematic review to evaluate its primary studies. It also provides a summary of the original authors’ conclusions regarding the overall quality or risk of bias found within their body of evidence. AHRQ, Agency for Healthcare Research and Quality; GRADE, Grading of Recommendations, Assessment, Development and Evaluation; PEDro, Physiotherapy Evidence Database; RCTs, Randomized Controlled Trials; RoB, Risk of Bias; ROBINS-I, Risk of Bias In Non-randomised Studiesof Interventions.

The authors’ conclusions on the quality of the primary evidence were mixed. Several reviews characterized the quality as generally “good” to “excellent”. For instance, Northey et al. (2017) reported a mean PEDro score of 6.7 (out of 10), indicating good quality. However, a substantial number of reviews described the evidence base as being of “fair” to “moderate” quality, or as having a “moderate” to “high” risk of bias. Commonly cited methodological weaknesses in the primary studies included inadequate reporting of randomization and allocation concealment, lack of blinding, and potential selection bias.

### 3.4 Methodological quality of included reviews

**FIGURE 2:**
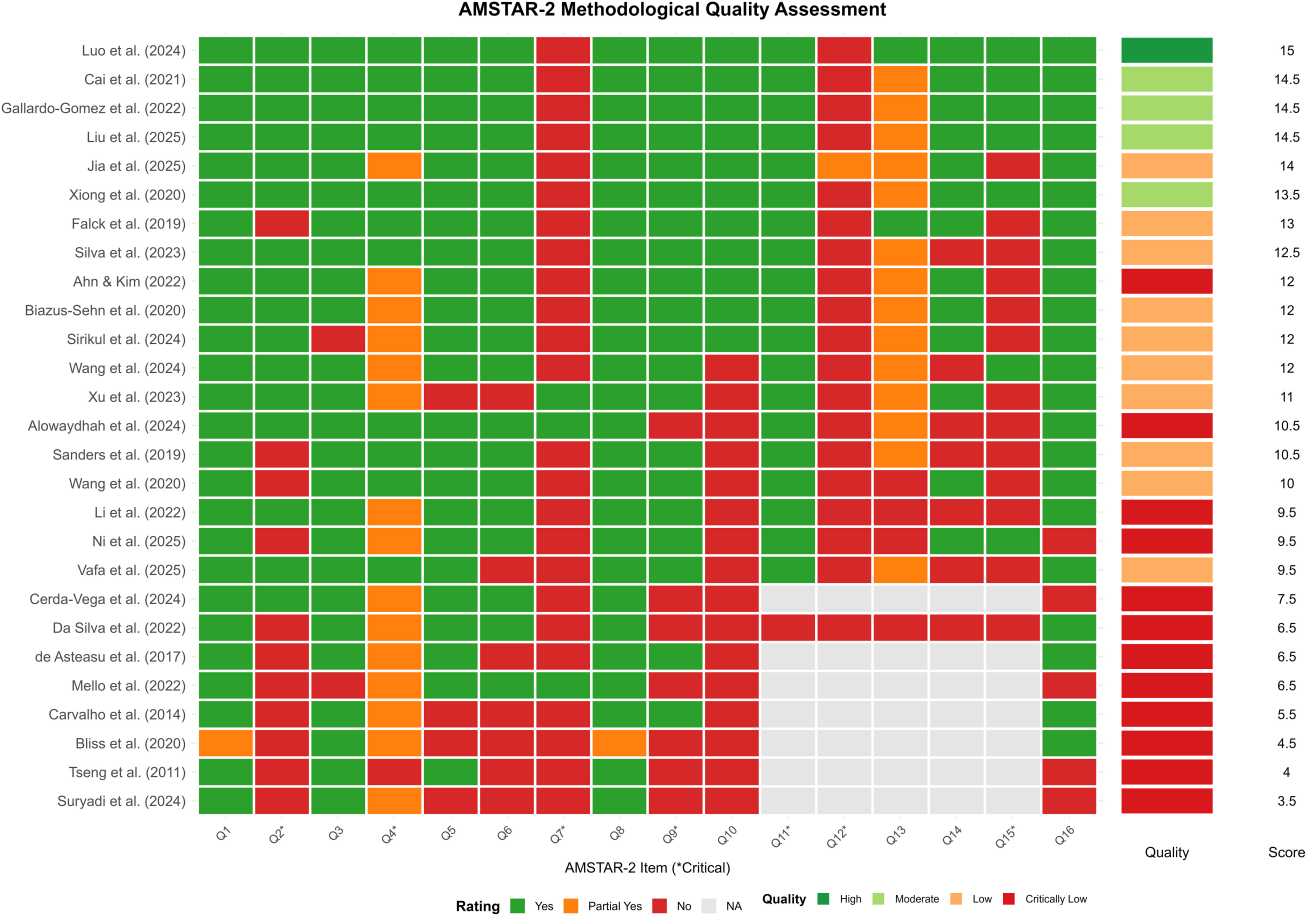
Methodological quality assessment of included systematic reviews using the AMSTAR-2 tool. The heat map summarizes the risk of bias assessment for each of the 27 included reviews across the 16 items of the AMSTAR-2 tool. Green indicates a “Yes” rating (item satisfied), yellow indicates a “Partial Yes” rating, and red indicates a “No” rating (item not satisfied), providing a visual overview of the methodological strengths and weaknesses of the evidence base.

The methodological quality of the included reviews was assessed using the AMSTAR-2 tool (Supplementary Table 2). Overall, the quality was highly variable. Based on the AMSTAR-2 criteria, one review was rated as “igh” quality, four as “Moderate” quality, 11 as “Low” quality, and 11 as “Critically Low” quality (Figure 2).

**FIGURE 3:**
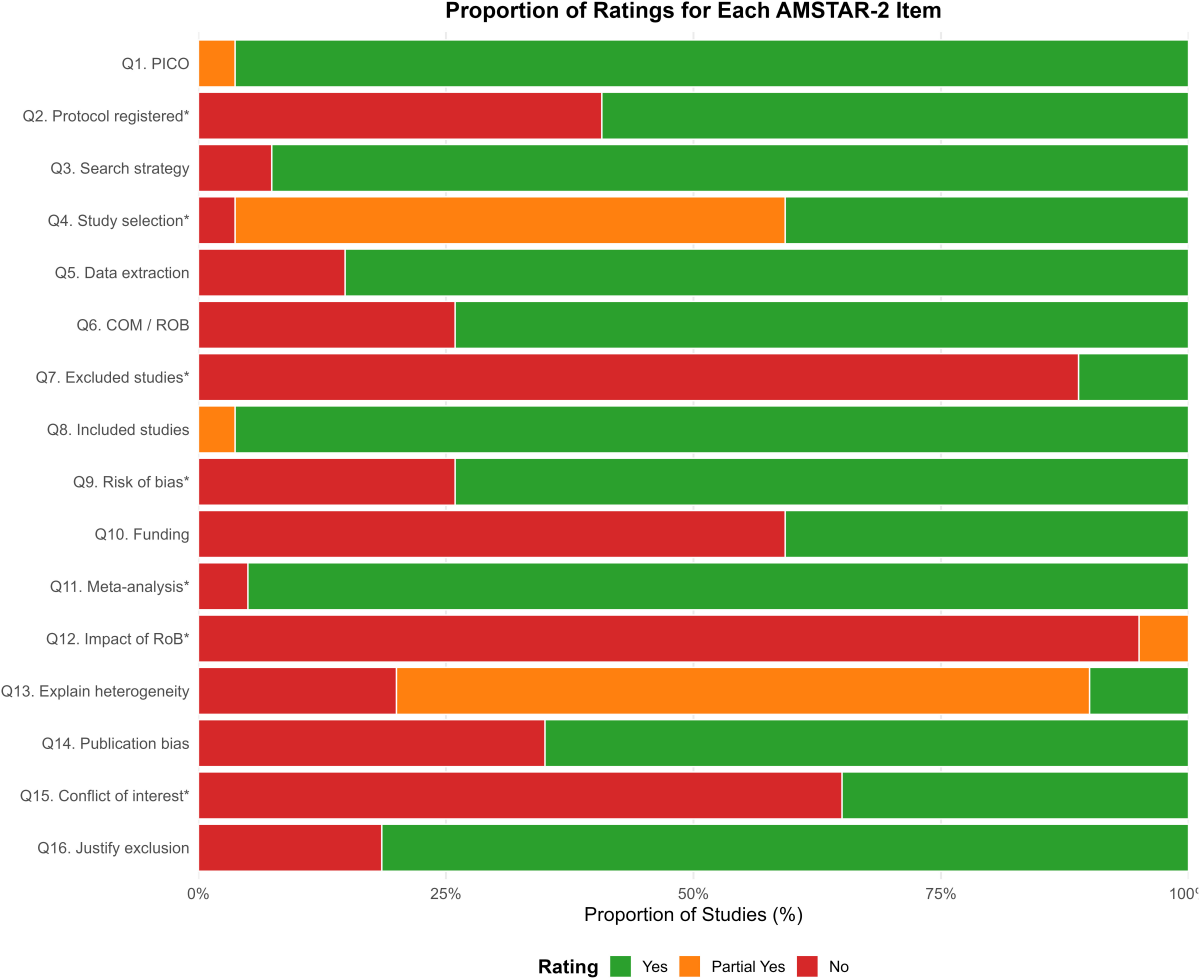
Forest plot of the pooled effect of multicomponent exercise on Global Cognitive Function. The plot displays the standardized mean difference (SMD) and 95% confidence interval (CI) for each individual systematic review assessing global cognitive function. The size of each square is proportional to the weight of the study in the meta-analysis. The diamond represents the overall pooled effect estimate, which shows a significant positive effect favoring the multicomponent exercise intervention.

Several critical methodological weaknesses were prevalent, leading to the downgrading of most reviews. The most common critical flaws included a failure to register a protocol a priori (Q2), not providing a list of excluded studies with justification (Q7), failing to discuss the likely impact of risk of bias on the results (Q12), and not adequately reporting on conflicts of interest (Q15) (Figure 3). These widespread limitations temper the confidence in the conclusions of many of the included reviews.

### 3.5 Synthesis of meta-analytic findings

**FIGURE 4:**
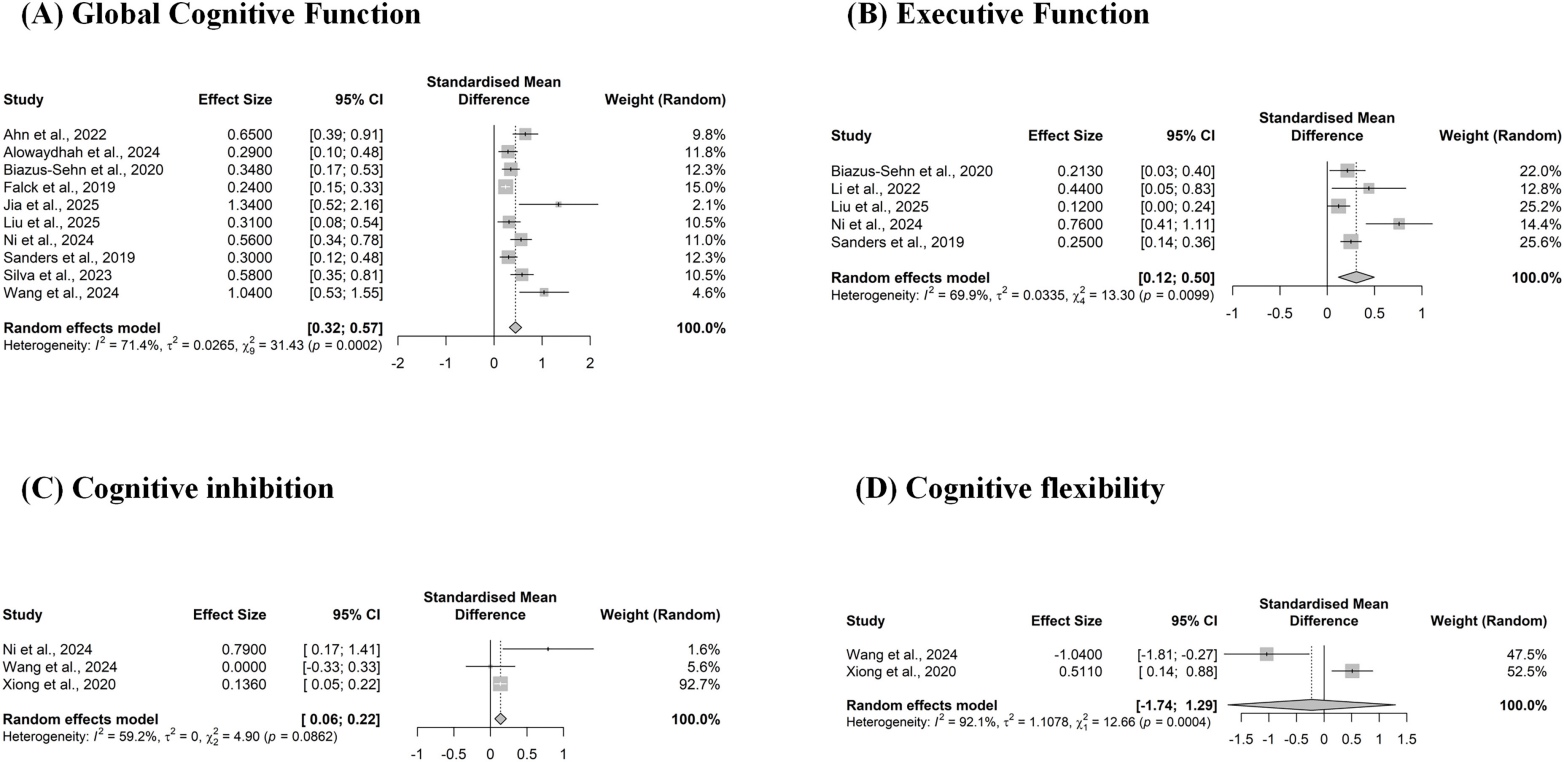
Forest plot of the pooled effect of multicomponent exercise on cognitive functions. (A) Global cognitive function; (B) Executive function; (C) Cognitive inhibition; and (D) Cognitive flexibility. The plot displays the standardized mean difference (SMD) and 95% confidence interval (CI) for each systematic review. The squares represent the effect estimate from each review, and the horizontal lines indicate the 95% CI. The diamond at the bottom of each subplot represents the overall pooled effect, indicating a significant benefit of multicomponent exercise.

The pooled effects of multicomponent exercise on various cognitive domains are summarized in Table 4. For global cognitive function (Figure 4A), the meta-analysis of 10 studies demonstrated a moderate and statistically significant positive effect [SMD = 0.45, 95% CI (0.32, 0.57), *p* < 0.001], though with substantial heterogeneity (*I*^2^ = 71.4%).

**TABLE 4:** Summary of findings table (base on GRADE).

Outcome	No. of studies (N)	Effect [95% CI]	Certainty of evidence (GRADE)	Comment
Global cognitive function	10	SMD = 0.45 [0.32, 0.57]	⊕⊕⊕O Moderate	The effect size is large and statistically significant. Downgraded by one level due to risk of bias in the original studies.
Global cognitive function (MD)	2	MD = 14.98 [12.47, 18.00]	⊕⊕OO Low	The effect size is large and significant. Downgraded by two levels for serious imprecision (very small number of studies) and risk of bias.
Executive function	5	SMD = 0.31 [0.12, 0.50]	⊕⊕OO Low	The effect size is large and significant. Downgraded by two levels for risk of bias and serious inconsistency (*I*^2^ = 69.9%).
Executive function (MD)	2	MD = 0.30 [0, 0.78]	⊕OOO Very Low	The effect is imprecise and the result is abnormal (extremely wide CI). Downgraded by three levels for serious imprecision, serious inconsistency (*I*^2^ = 87.7%), and risk of bias.
Attention/processing speed	4	SMD = 0.19 [-0.12, 0.50]	⊕OOO Very Low	The effect size is large and significant. Downgraded by three levels for risk of bias, very serious inconsistency (*I*^2^ = 83.5%), and imprecision (small number of studies).
Memory (overall)	3	SMD = 0.18 [0.06, 0.29]	⊕⊕OO Low	The effect size is large and significant with low heterogeneity. Downgraded by two levels for risk of bias and serious imprecision due to the very small number of studies.
Immediate memory	2	SMD = 0.06 [-0.06, 0.18]	⊕⊕OO Low	The effect size is large and significant with no heterogeneity. Downgraded by two levels for risk of bias and serious imprecision due to the very small number of studies.
Delayed memory	2	SMD = 0.11 [-0.07, 0.29]	⊕⊕OO Low	The effect size is large and significant with low heterogeneity. Downgraded by two levels for risk of bias and serious imprecision due to the very small number of studies.
Verbal memory	4	SMD = 0.33 [0.19, 0.46]	⊕⊕OO Low	The effect size is large and significant with no heterogeneity. Downgraded by two levels for imprecision (small number of studies) and risk of bias.
Working memory	4	SMD = 0.19 [-0.01, 0.38]	⊕⊕OO Low	The effect size is large and significant. Downgraded by two levels for risk of bias and moderate inconsistency (*I*^2^ = 61.1%).
Cognitive inhibition	3	SMD = 0.14 [0.06, 0.22]	⊕⊕OO Low	The effect size is large and significant. Downgraded by two levels for imprecision, risk of bias, and moderate inconsistency (*I*^2^ = 59.2%).
Cognitive flexibility	2	SMD = -0.23 [-1.74, 1.29]	⊕OOO Very low	The effect is large but very imprecise (extremely wide CI). Downgraded by three levels for very serious imprecision, very serious inconsistency (*I*^2^ = 92.1%), and risk of bias.

This table presents a summary of the overall certainty of evidence for the main cognitive outcomes, as assessed using the GRADE framework. For each outcome, the table specifies the number of studies contributing to the pooled effect, the effect size and 95% confidence interval, the final GRADE rating, and a comment explaining the rationale for any downgrading of the evidence. Certainty of Evidence Ratings: ⊕⊕⊕⊕ High; ⊕⊕⊕O Moderate; ⊕⊕OO Low; ⊕OOO Very low. CI, Confidence Interval; *I*^2^, I-squared statistic for heterogeneity; MD, Mean Difference; N, Number of Studies; SMD, Standardized Mean Difference.

Regarding executive function (Figure 4B), the pooled analysis of 5 studies showed a large, significant benefit [SMD = 0.31, 95% CI (0.12, 0.50), *p* = 0.002], also with substantial heterogeneity (*I*^2^ = 69.9%). Within this domain, cognitive inhibition (Figure 4C) showed a small but significant effect (SMD = 0.14), while the effect on cognitive flexibility (Figure 4D) was not statistically significant [95% CI (-1.74, 1.29)].

**FIGURE 5:**
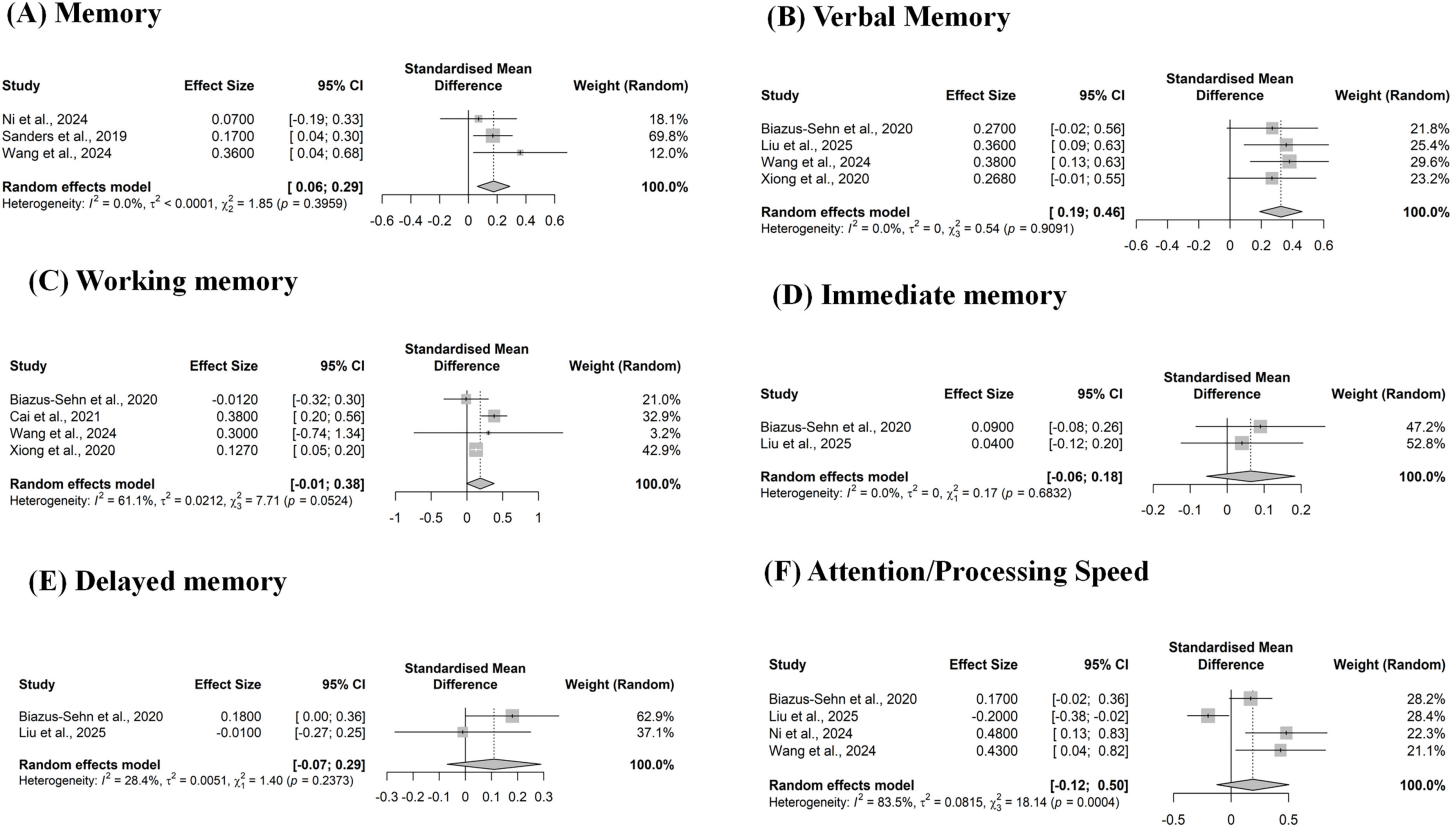
Forest plot of the pooled effect of multicomponent exercise on Memory. (A) Memory; (B) Verbal Memory; (C) Working Memory; (D) Immediate Memory; (E) Delayed Memory; (F) Attention/Processing Speed. The plot displays the standardized mean difference (SMD) and 95% confidence interval (CI) for each systematic review assessing memory outcomes. The size of the square reflects the study’s weight. The diamond represents the overall pooled effect, showing a significant positive effect of multicomponent exercise on memory.

The intervention also showed significant benefits across multiple memory domains. The overall analysis for memory (3 studies) yielded a small, significant effect [SMD = 0.18, 95% CI (0.06, 0.29)] (Figure 5A). The effect was particularly strong for verbal memory (four studies; SMD = 0.33) (Figure 5B). However, analyses for working memory (Figure 5C), immediate memory (Figure 5D), and delayed memory (Figure 5E) did not reach statistical significance, as their confidence intervals crossed zero.

For attention and processing speed (Figure 5F), the analysis of 4 studies indicated a large pooled non-significant pooled effect [SMD = 0.19, 95% CI [-0.12, 0.50)] associated with considerable heterogeneity (*I*^2^ = 83.5%).

### 3.6 Subgroup analysis by baseline cognitive status

**FIGURE 6:**
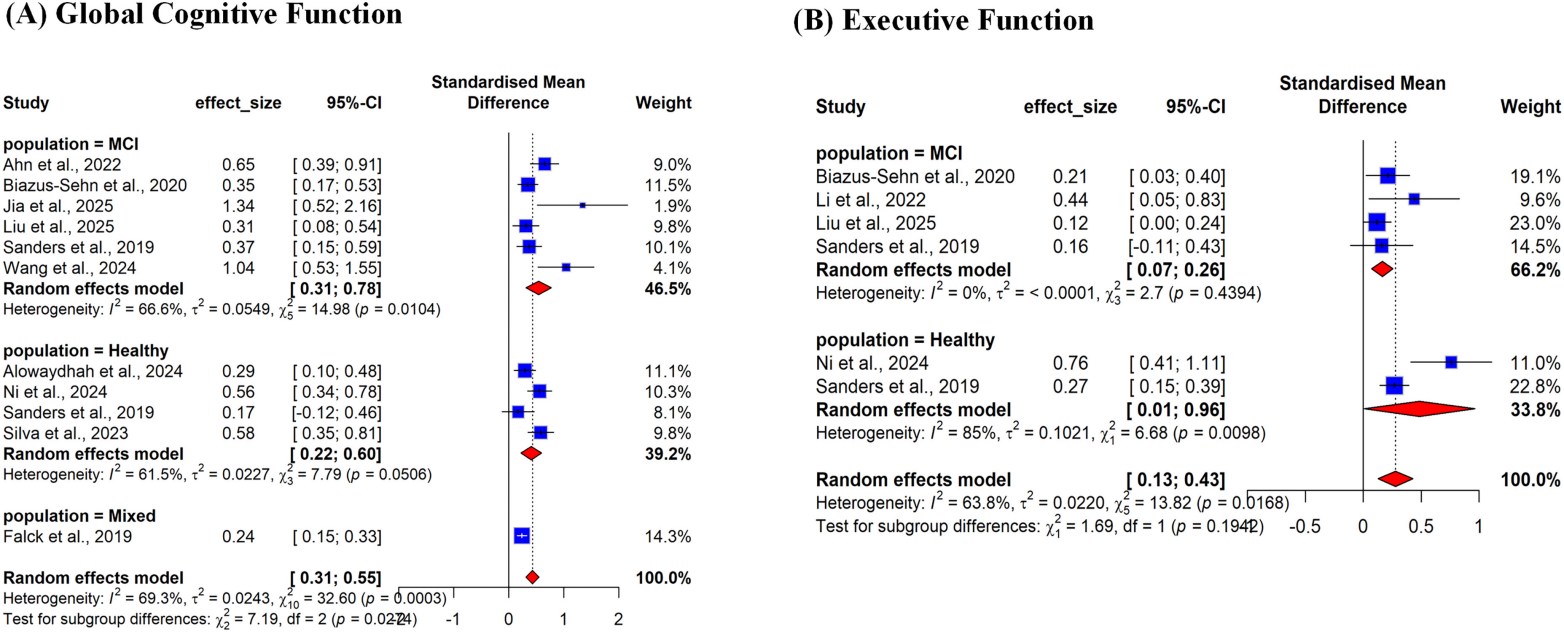
Subgroup analysis of the effect of multicomponent exercise based on participants’ baseline cognitive status. (A) Global Cognitive Function; (B) Executive Function. The forest plot displays the pooled standardized mean difference (SMD) stratified by participant subgroups, such as “Cognitively Healthy” and “Mild Cognitive Impairment (MCI)”. Eachdiamond represents the overall pooled effect estimate and its 95% confdence interval for that specific subgroup. The ploti used to visualy inspectfor differences in the intervention’s effect across different populations and to test for subgroup differences.

Subgroup analyses were conducted to investigate the influence of baseline cognitive status on the intervention’s effectiveness (Supplementary Table 3). For global cognitive function (Figure 6A), significant moderate effects were found in both the MCI subgroup (6 studies; SMD = 0.54) and the healthy subgroup (4 studies; SMD = 0.41), with substantial heterogeneity present in both analyses. We notably did not generate a quantitative forest plot for the “Frailty” subgroup due to the limited number of reviews reporting compatible Standardized Mean Differences (SMD) for this specific population. However, individual reviews focusing on frail older adults (e.g., Sirikul et al., 2024; Wang et al., 2020) consistently reported positive outcomes, with effect sizes generally ranging from small to moderate (SMD ∼ 0.18–0.33), aligning with the trends observed in the Healthy and MCI subgroups. For executive function (Figure 6B), both subgroups showed significant effects, with a small effect observed in participants with MCI (4 studies; SMD = 0.16) and a moderate effect in healthy participants (two studies; SMD = 0.49).

### 3.7 Certainty of evidence (GRADE assessment)

A GRADE assessment was performed to evaluate the overall confidence in the pooled effect estimates (Table 4). Based on the GRADE assessment, the certainty of evidence varied across domains. Global cognitive function was the only outcome supported by “Moderate” certainty evidence; it was downgraded by one level primarily due to the risk of bias in the underlying primary studies. In contrast, the certainty of evidence for Executive Function, Memory (including overall, verbal, immediate, delayed, and working memory), and Cognitive Inhibition was rated as “Low.” These outcomes were typically downgraded due to a combination of risk of bias and inconsistency (heterogeneity) or imprecision. Finally, the evidence for Attention/Processing Speed and Cognitive Flexibility was graded as “Very Low,” reflecting serious limitations in inconsistency (*I*^2^ > 80%) and imprecision (wide confidence intervals).

### 3.8 Sensitivity analysis

The results of the sensitivity analysis demonstrated that the overall findings were robust. As shown in Supplementary Figures 1–7, the sequential removal of each individual study did not substantially alter the magnitude or direction of the pooled effect sizes for global cognitive function, executive function, or memory. In all iterations, the recalculated pooled effect estimates and their 95% confidence intervals remained consistent with the primary analysis, indicating that no single systematic review disproportionately influenced the overall results. This strengthens the confidence in the conclusion that multicomponent exercise has a positive effect on cognitive function in older adults.

## 4 Discussion

### 4.1 Summary of principal findings

This umbrella review synthesized evidence from 27 systematic reviews and meta-analyses, providing a comprehensive assessment of the effects of multicomponent exercise (MCE) on cognitive function in older adults. To visually summarize the core findings of this umbrella review and their hierarchy of evidence, a graphical abstract is presented. The findings overwhelmingly demonstrate that MCE is an effective strategy for improving cognition across a spectrum of older populations. The meta-analytic results revealed statistically significant, moderate positive effects on global cognitive function and executive function. While MCE also conferred a benefit for overall memory (specifically verbal memory), the analyses for working memory, delayed memory, and attention/processing speed did not reach statistical significance, suggesting that the efficacy of MCE may vary across specific cognitive sub-domains. Subgroup analyses further indicated that these benefits are present in both cognitively healthy individuals and those with Mild Cognitive Impairment (MCI).

However, this positive conclusion is moderated by a critical appraisal of the evidence base. Despite the consistency of the findings, our methodological quality assessment using AMSTAR-2 revealed that the majority of the included reviews were of “Low” to “Critically Low” quality. Consequently, the certainty of the evidence for most cognitive outcomes, as assessed by the GRADE framework, was judged to be “Low” to “Very Low,” with only global cognitive function achieving a “Moderate” rating. This discrepancy between the magnitude of the effect and the quality of the evidence is a central theme of our findings.

### 4.2 Interpretation and comparison with existing literature

The consistent finding that MCE enhances Global Cognitive Function and Executive Function aligns with the “Synergistic Hypothesis” of exercise adaptation. Unlike single-mode interventions, MCE targets the cardiovascular, musculoskeletal, and neuromuscular systems simultaneously. Aerobic components are known to increase cerebral blood flow and Brain-Derived Neurotrophic Factor (BDNF) levels, promoting vascular health and neurogenesis (Suzuki et al., 2013; Rondão et al., 2022; Venegas-Sanabria et al., 2022). Concurrently, resistance training stimulates the release of peripheral myokines (e.g., irisin, IGF-1), which can cross the blood-brain barrier to support synaptic plasticity (Rondão et al., 2022). By combining these modalities, MCE likely induces a “multi-pathway” stimulation that offers superior neuroprotection compared to aerobic or resistance training alone.

However, our meta-analytic synthesis also revealed a divergence in efficacy across cognitive domains. While Global Cognition and Executive Function showed significant moderate improvements, specific sub-domains such as Working Memory, Delayed Memory, and Attention/Processing Speed did not reach statistical significance. This finding can be interpreted through the “Specificity Principle” of training(Hawley, 2008). Executive functions, which involve planning and inhibition, are inherently engaged during MCE (e.g., learning complex movement sequences, coordinating limbs). In contrast, domains like Working Memory and Processing Speed may require more specific, high-load cognitive demands—such as dual-tasking with high interference—to show robust improvements (Wollesen et al., 2020; Park et al., 2021; Ludyga et al., 2023). The high heterogeneity observed in these non-significant domains suggests that standard MCE programs, which primarily focus on physical outcomes, may not consistently provide the specific cognitive load required to boost these particular faculties.

Furthermore, our subgroup analyses suggest that MCE is a versatile intervention, demonstrating efficacy in both cognitively healthy individuals and those with MCI. The comparable effect sizes on global cognition in both groups underscore MCE’s dual role: as a preventative strategy to build cognitive reserve in healthy aging and as a therapeutic intervention to slow cognitive decline in at-risk populations. The evidence for frail individuals was slightly more ambiguous but still leaned positive, indicating the need for careful adaptation of programs for this vulnerable group.

The substantial statistical heterogeneity observed in our analyses (*I*^2^ > 70% for several outcomes) warrants careful interpretation regarding the “causes of heterogeneity.” Our subgroup analysis (Figure 6) indicates that baseline cognitive status is one contributing factor; for example, distinguishing between participants with MCI and healthy older adults partially explains the variance in treatment response. However, considerable heterogeneity remains even within subgroups. This is likely attributable to the high variability in MCE protocols across the primary studies—specifically differences in the FITT principles (Frequency, Intensity, Time, and Type). The included reviews synthesized programs ranging from moderate to high intensity, with session durations varying from 30 to 90 min, and utilizing diverse equipment (e.g., machines vs. elastic bands). Furthermore, the “measurement heterogeneity” poses a challenge, as different neuropsychological tests (e.g., Stroop test vs. Trail Making Test) were often pooled under broad domains like “Executive Function.” Despite this variability in the magnitude of the effect, the direction of the findings remains consistently positive across diverse protocols, suggesting that the benefit of MCE is robust to protocol variations.

### 4.3 Strengths and limitations

The primary strength of this umbrella review is its comprehensive and systematic approach. By synthesizing a large and recent body of evidence from 27 reviews, we provide a high-level, panoramic view of the current state of the science. The rigorous dual assessment of evidence quality—evaluating the methodological quality of the reviews themselves with AMSTAR-2 and assessing the certainty of the outcomes with GRADE—provides a transparent and critical appraisal of the evidence base, which is a significant contribution to the literature.

However, several limitations must be acknowledged. First, as an umbrella review, our findings are inherently constrained by the quality and reporting of the included systematic reviews and the primary studies they contain. The “Low” to “Critically Low” AMSTAR-2 ratings for most reviews indicate that methodological flaws in the evidence base are common, which was the primary reason for downgrading the certainty of evidence in our GRADE assessment. Second, a specific limitation inherent to umbrella reviews is the overlap of primary studies across the included systematic reviews, which can lead to double-counting and potentially inflated statistical precision. To mitigate interpretation errors arising from this issue, we took the precaution of conducting a leave-one-out sensitivity analysis (see section 3.8 and Supplementary Figures 1–7). This analysis demonstrated that the removal of any single systematic review did not substantially alter the magnitude or significance of the pooled effect sizes for global cognitive function or executive function. This stability suggests that our conclusions are robust and are not disproportionately driven by a specific subset of duplicated primary trials. Nevertheless, readers should prioritize the consistent direction of the findings across diverse reviews over the precise width of the confidence intervals. Finally, the high heterogeneity across studies, while an important finding, limits our ability to make specific recommendations regarding the optimal design and dosage of MCE protocols.

Crucially, the impact of the predominantly “Low” evidence quality ratings (assessed via GRADE) must be interpreted with nuance. This rating does not necessarily imply that the intervention is ineffective; rather, it reflects limited confidence in the precise magnitude of the effect estimate. In exercise science, achieving “High” certainty is inherently challenging because double-blinding (blinding participants to the intervention) is impossible, which automatically triggers specific risk-of-bias penalties in tools like AMSTAR-2 and GRADE. Therefore, while the “Low” quality indicates that future rigorous research (e.g., with blinded outcome assessors) might change the estimated effect size, it should not preclude the clinical implementation of MCE. Given the consistent positive direction of the findings across 27 reviews and the low risk of adverse events associated with exercise, the current evidence remains sufficient to support MCE as a pragmatic public health strategy.

### 4.4 Implications for clinical practice and public health

Despite the limitations in evidence quality, the consistency and magnitude of the observed effects have clear implications. The findings provide robust support for clinicians and public health bodies to confidently recommend multicomponent exercise to older adults to maintain or improve cognitive function.

1. For Prevention and Treatment: MCE should be considered a core component of health promotion strategies for cognitively healthy older adults and a frontline, non-pharmacological intervention for individuals with MCI.
2. Program Design: While an optimal “dose” cannot be definitively prescribed, effective programs should integrate aerobic, resistance, and balance training. The inclusion of components that require motor learning and cognitive engagement appears to be particularly beneficial.
3. Accessibility: MCE programs are highly adaptable and can be tailored to various functional levels, making them a feasible and scalable public health strategy that can be implemented in community centers, clinics, and long-term care facilities.

### 4.5 Implications for future research

This review highlights several critical gaps and directions for future research. High-Quality Primary Trials: There is a pressing need for more large-scale, high-quality RCTs with robust methodology, including a priori protocol registration, adequate blinding of outcome assessors, and transparent reporting according to CONSORT guidelines.

1. Standardization: To reduce heterogeneity and enable more meaningful comparisons, the field would benefit from establishing a core outcome set for cognitive assessment in exercise trials and standardizing the definition and reporting of MCE interventions based on FITT principles (Frequency, Intensity, Time, Type).

2. Head-to-Head Comparisons: Future trials should move beyond comparing MCE to non-exercise controls and conduct head-to-head comparisons of different MCE protocols to dismantle the “black box” and identify which combinations, doses, and progressions of exercise are most effective.

3. Mechanistic Studies: Research incorporating neuroimaging and biomarkers is needed to further elucidate the underlying neurobiological mechanisms through which MCE confers its cognitive benefits.

4. Long-term Follow-up: Longer-term studies are required to determine the sustainability of cognitive improvements and the potential for MCE to delay the incidence of dementia.

## 5 Conclusion

In conclusion, this umbrella review confirms that multicomponent exercise is a powerful non-pharmacological strategy for enhancing cognitive function in a broad spectrum of older adults. The evidence strongly supports its positive effects on global cognition, executive function, and, and overall memory performance. However, evidence for improvements in specific sub-domains, such as working memory and processing speed, remains inconclusive and highly heterogeneous. Consequently, the certainty of this evidence is frequently limited by the methodological weaknesses and heterogeneity of the primary literature. These findings underscore the urgent need for promoting multicomponent exercise in clinical and community settings for general cognitive health, while simultaneously calling for more rigorous, standardized, and mechanistic research to optimize intervention protocols for specific cognitive domains.

## Data Availability

The original contributions presented in this study are included in this article/Supplementary material, further inquiries can be directed to the corresponding authors.
