# Correction: The efficacy versus evidence quality of multicomponent exercise for cognitive health in older adults: an umbrella review

**DOI:** 10.3389/fnagi.2026.1798753

**Published:** 2026-02-23

**Authors:** Weibao Liang, Chuannan Liu, Xujie Yan, Shuting Xu, Jianmin Dai, Wenbai Huang

**Affiliations:** 1 Guangdong Provincial Key Laboratory of Speed Capability Research, Su Bingtian Center for Speed Research and Training, School of Physical Education, Jinan University, Guangzhou, China; 2 School of Physical Education and Health, Zunyi Medical University, Zunyi, China; 3 School of Physical Education and Sports Science, Hengyang Normal University, Hengyang, China; 4 The First Affiliated Hospital of Jinan University, Guangzhou, China; 5 College of Sports Science, Kyungnam University, Changwon, Republic of Korea

**Keywords:** multicomponent exercise, cognitive function, older adults, physical activity, executive function

In the published article, the title was erroneously given as: “The efficacy versus evidence quality of multicompomnent exercise for cognitive health in older adults: an umbrella review” in both the main title and citation section. Instead, the title of the article should have been: “The efficacy versus evidence quality of multicomponent exercise for cognitive health in older adults: an umbrella review”.

The original version of this article has been updated.

