# Supplementary material for: The efficacy versus evidence quality of multicomponent exercise for cognitive health in older adults: an umbrella review
Source: Front Aging Neurosci. 2025 Dec 15;17:1719179. doi: 10.3389/fnagi.2025.1719179 (PMC12745398; doi:10.3389/fnagi.2025.1719179)

# Supplemental Online Content

**Table S1. Search Strategy.**

**Table S2. Table S2. The methodological quality of the included reviews was assessed using the AMSTAR-2 tool.**

**Table S3. Summary of subgroup analyses.**

**Figures S1-S7. Leave-one-out sensitivity analyses.**

# Table S1. Search Strategy.

Pubmed

| #1 | "sprint interval training"[Title/Abstract] OR "sprint interval exercise"[Title/Abstract] OR "sprint intermittent training"[Title/Abstract] OR "sprint training"[Title/Abstract] OR "sprint-interval training"[Title/Abstract] OR "High-Intensity Interval Training"[Mesh] OR "High intensity intermittent training"[Title/Abstract] OR "High-intensity intermittent training"[Title/Abstract] OR "Interval training"[Title/Abstract] OR "interval exercise"[Title/Abstract] OR "HIIT"[Title/Abstract] OR "high intensity exercise"[Title/Abstract] OR "high intensity aerobic interval training"[Title/Abstract] OR "all-out exercise training"[Title/Abstract] OR "all-out training"[Title/Abstract] OR "all-out interval training"[Title/Abstract] OR "Supramaximal interval training"[Title/Abstract] OR "Wingate training"[Title/Abstract] OR "maximal intensity"[Title/Abstract] |
| --- | --- |
| #2 | "Diabetes Mellitus, Type 2"[Mesh] OR "Prediabetic State"[Mesh] OR "Insulin Resistance"[Mesh] OR "Metabolic Syndrome"[Mesh] OR "type 2 diabetes"[tiab] OR "T2DM"[tiab] OR "prediabetes"[tiab] OR "metabolic syndrome"[tiab] OR "insulin resistance"[tiab] OR "glycemic control"[tiab] OR "glucose tolerance"[tiab] OR overweight[tiab] OR obese[tiab] ) |
| #3 | "randomized controlled trial"[pt] OR "controlled clinical trial"[pt] OR randomized[tiab] OR placebo[tiab] OR "drug therapy"[sh] OR randomly[tiab] OR trial[tiab] OR groups[tiab] |
| #4 | #1 AND #2 AND #3 |

Web of Science

| #1 | TS=("sprint interval training" OR "sprint interval exercise" OR "Sprint intermittent training" OR "sprint training" OR "sprint-interval training" OR "High-Intensity Interval Training" OR "High intensity intermittent training" OR "High-intensity intermittent training" OR "Interval training" OR "interval exercise" OR "HIIT" OR "high intensity exercise" OR "high intensity aerobic interval training" OR "all-out exercise training" OR "all-out training" OR "all-out interval training" OR "Supramaximal interval training" OR "Wingate training" OR "maximal intensity") |
| --- | --- |
| #2 | TS=("type 2 diabetes" OR T2DM OR prediabetes OR "metabolic syndrome" OR "insulin resistance" OR "glycemic control" OR "glucose tolerance" OR overweight OR obese) |
| #3 | TS=(randomized OR randomised OR placebo OR "controlled trial" OR trial OR groups) |
| #4 | #1 AND #2 AND #3 |

SPORTDiscus

| S1 | DE "HIGH intensity interval training" OR DE "SPRINT training" OR DE "INTERVAL training" OR TI ( "sprint interval training" OR "sprint interval exercise" OR "Sprint intermittent training" OR "sprint training" OR "sprint-interval training" OR "High-Intensity Interval Training" OR "High intensity intermittent training" OR "High-intensity intermittent training" OR "Interval training" OR "interval exercise" OR "HIIT" OR "high intensity exercise" OR "high intensity aerobic interval training" OR "all-out exercise training" OR "all-out training" OR "all-out interval training" OR "Supramaximal interval training" OR "Wingate training" OR "maximal intensity" ) OR AB ( "sprint interval training" OR "sprint interval exercise" OR "Sprint intermittent training" OR "sprint training" OR "sprint-interval training" OR "High-Intensity Interval Training" OR "High intensity intermittent training" OR "High-intensity intermittent training" OR "Interval training" OR "interval exercise" OR "HIIT" OR "high intensity exercise" OR "high intensity aerobic interval training" OR "all-out exercise training" OR "all-out training" OR "all-out interval training" OR "Supramaximal interval training" OR "Wingate training" OR "maximal intensity" ) |
| --- | --- |
| S2 | DE "TYPE II diabetes" OR DE "PREDIABETES" OR DE "INSULIN resistance" OR DE "METABOLIC syndrome" OR TI ( "type 2 diabetes" OR T2DM OR prediabetes OR "insulin resistance" OR "glycemic control" OR overweight OR obese ) OR AB ( "type 2 diabetes" OR T2DM OR prediabetes OR "insulin resistance" OR "glycemic control" OR overweight OR obese ) |
| S3 | TI ( randomized OR randomised OR placebo OR "controlled trial" OR trial OR groups ) OR AB ( randomized OR randomised OR placebo OR "controlled trial" OR trial OR groups ) |
| S4 | S1 AND S2 AND S3 |

Cochrane Library

| #1 | [mh "High-Intensity Interval Training"] OR "sprint interval training":ti,ab,kw OR "sprint interval exercise":ti,ab,kw OR "Sprint intermittent training":ti,ab,kw OR "sprint training":ti,ab,kw OR "sprint-interval training":ti,ab,kw OR "High intensity intermittent training":ti,ab,kw OR "High-intensity intermittent training":ti,ab,kw OR "Interval training":ti,ab,kw OR "interval exercise":ti,ab,kw OR "HIIT":ti,ab,kw OR "high intensity exercise":ti,ab,kw OR "high intensity aerobic interval training":ti,ab,kw OR "all-out exercise training":ti,ab,kw OR "all-out training":ti,ab,kw OR "all-out interval training":ti,ab,kw OR "Supramaximal interval training":ti,ab,kw OR "Wingate training":ti,ab,kw OR "maximal intensity":ti,ab,kw |
| --- | --- |
| #2 | [mh "Diabetes Mellitus, Type 2"] OR [mh "Prediabetic State"] OR [mh "Insulin Resistance"] OR [mh "Metabolic Syndrome"] |
| #3 | #1 AND #2 |

Embase

| #1 | 'high intensity interval training'/exp OR 'sprint interval training':ti,ab,kw OR 'sprint interval exercise':ti,ab,kw OR 'sprint intermittent training':ti,ab,kw OR 'sprint training':ti,ab,kw OR 'sprint-interval training':ti,ab,kw OR 'high intensity intermittent training':ti,ab,kw OR 'high-intensity intermittent training':ti,ab,kw OR 'interval training':ti,ab,kw OR 'interval exercise':ti,ab,kw OR 'hiit':ti,ab,kw OR 'high intensity exercise':ti,ab,kw OR 'high intensity aerobic interval training':ti,ab,kw OR 'all-out exercise training':ti,ab,kw OR 'all-out training':ti,ab,kw OR 'all-out interval training':ti,ab,kw OR 'supramaximal interval training':ti,ab,kw OR 'wingate training':ti,ab,kw OR 'maximal intensity':ti,ab,kw |
| --- | --- |
| #2 | 'type 2 diabetes mellitus'/exp OR 'prediabetes'/exp OR 'insulin resistance'/exp OR 'metabolic syndrome'/exp OR 'type 2 diabetes':ti,ab OR 'T2DM':ti,ab OR 'prediabetes':ti,ab OR 'metabolic syndrome':ti,ab OR 'insulin resistance':ti,ab OR 'glycemic control':ti,ab OR 'glucose tolerance':ti,ab OR 'overweight':ti,ab OR 'obese':ti,ab |
| #3 | 'randomized controlled trial'/exp OR 'controlled clinical trial'/exp OR 'randomization'/exp OR 'placebo'/exp OR 'randomly':ti,ab OR 'trial':ti,ab OR 'groups':ti,ab |
| #4 | #1 AND #2 AND #3 |

Scopus

|  | TITLE-ABS-KEY ( "sprint interval training" OR "sprint interval exercise" OR "Sprint intermittent training" OR "sprint training" OR "sprint-interval training" OR "High-Intensity Interval Training" OR "High intensity intermittent training" OR "High-intensity intermittent training" OR "Interval training" OR "interval exercise" OR "HIIT" OR "high intensity exercise" OR "high intensity aerobic interval training" OR "all-out exercise training" OR "all-out training" OR "all-out interval training" OR "Supramaximal interval training" OR "Wingate training" OR "maximal intensity" ) |
| --- | --- |
| AND |  |
|  | TITLE-ABS-KEY("type 2 diabetes" OR T2DM OR prediabetes OR "metabolic syndrome" OR "insulin resistance" OR "glycemic control" OR "glucose tolerance" OR overweight OR obese) |
| AND |  |
|  | TITLE-ABS-KEY(randomized OR randomised OR placebo OR "controlled trial" OR trial OR groups) |

**Table S2. The methodological quality of the included reviews was assessed using the AMSTAR-2 tool.**

| **Study** | **Q1** | **Q2** | **Q3** | **Q4** | **Q5** | **Q6** | **Q7** | **Q8** | **Q9** | **Q10** | **Q11** | **Q12** | **Q13** | **Q14** | **Q15** | **Q16** | **Score** | **Quality** |
| --- | --- | --- | --- | --- | --- | --- | --- | --- | --- | --- | --- | --- | --- | --- | --- | --- | --- | --- |
| Ahn & Kim (2022) | Y | Y | Y | PY | Y | Y | N | Y | Y | Y | Y | N | PY | Y | N | Y | 12 | Critically Low |
| Alowaydhah et al. (2024) | Y | Y | Y | Y | Y | Y | Y | Y | N | N | Y | N | PY | N | N | Y | 10.5 | Critically Low |
| Biazus-Sehn et al. (2020) | Y | Y | Y | PY | Y | Y | N | Y | Y | Y | Y | N | PY | Y | N | Y | 12 | Low |
| Bliss et al. (2020) | PY | N | Y | PY | N | N | N | PY | N | N | NA | NA | NA | NA | NA | Y | 4.5 | Critically Low |
| Cai et al. (2021) | Y | Y | Y | Y | Y | Y | N | Y | Y | Y | Y | N | PY | Y | Y | Y | 14.5 | Moderate |
| Carvalho et al. (2014) | Y | N | Y | PY | N | N | N | Y | Y | N | NA | NA | NA | NA | NA | Y | 5.5 | Critically Low |
| Cerda-Vega et al. (2024) | Y | Y | Y | PY | Y | Y | N | Y | N | N | NA | NA | NA | NA | NA | N | 7.5 | Critically Low |
| Da Silva et al. (2022) | Y | N | Y | PY | Y | Y | N | Y | N | N | N | N | N | N | N | Y | 6.5 | Critically Low |
| de Asteasu et al. (2017) | Y | N | Y | PY | Y | N | N | Y | Y | N | NA | NA | NA | NA | NA | Y | 6.5 | Critically Low |
| Falck et al. (2019) | Y | N | Y | Y | Y | Y | N | Y | Y | Y | Y | N | Y | Y | N | Y | 13 | Low |
| Gallardo-Gomez et al. (2022) | Y | Y | Y | Y | Y | Y | N | Y | Y | Y | Y | N | PY | Y | Y | Y | 14.5 | Moderate |
| Jia et al. (2025) | Y | Y | Y | PY | Y | Y | N | Y | Y | Y | Y | PY | PY | Y | N | Y | 14 | Low |
| Li et al. (2022) | Y | Y | Y | PY | Y | Y | N | Y | Y | N | Y | N | N | N | N | Y | 9.5 | Critically Low |
| Liu et al. (2025) | Y | Y | Y | Y | Y | Y | N | Y | Y | Y | Y | N | PY | Y | Y | Y | 14.5 | Moderate |
| Luo et al. (2024) | Y | Y | Y | Y | Y | Y | N | Y | Y | Y | Y | N | Y | Y | Y | Y | 15 | High |
| Mello et al. (2022) | Y | N | N | PY | Y | Y | Y | Y | N | N | NA | NA | NA | NA | NA | N | 6.5 | Critically Low |
| Ni et al. (2025) | Y | N | Y | PY | Y | Y | N | Y | Y | N | Y | N | N | Y | Y | N | 9.5 | Critically Low |
| Sanders et al. (2019) | Y | N | Y | Y | Y | Y | N | Y | Y | N | Y | N | PY | N | N | Y | 10.5 | Low |
| Silva et al. (2023) | Y | Y | Y | Y | Y | Y | N | Y | Y | Y | Y | N | PY | N | N | Y | 12.5 | Low |
| Sirikul et al. (2024) | Y | Y | N | PY | Y | Y | N | Y | Y | Y | Y | N | PY | Y | N | Y | 12 | Low |
| Suryadi et al. (2024) | Y | N | Y | PY | N | N | N | Y | N | N | NA | NA | NA | NA | NA | N | 3.5 | Critically Low |
| Tseng et al. (2011) | Y | N | Y | N | Y | N | N | Y | N | N | NA | NA | NA | NA | NA | N | 4 | Critically Low |
| Vafa et al. (2025) | Y | Y | Y | Y | Y | N | N | Y | Y | N | Y | N | PY | N | N | Y | 9.5 | Low |
| Wang et al. (2020) | Y | N | Y | Y | Y | Y | N | Y | Y | N | Y | N | N | Y | N | Y | 10 | Low |
| Wang et al. (2024) | Y | Y | Y | PY | Y | Y | N | Y | Y | N | Y | N | PY | N | Y | Y | 12 | Low |
| Xiong et al. (2020) | Y | Y | Y | Y | Y | Y | N | Y | Y | Y | Y | N | PY | Y | Y | Y | 13.5 | Moderate |
| Xu et al. (2023) | Y | Y | Y | PY | N | N | Y | Y | Y | N | Y | N | PY | Y | N | Y | 11 | Low |

**Table S3. Summary of subgroup analyses.**

| **Outcomes** | **Subgroups** | **No.Studies** | **SMD(95%CI)** | **P** | **I²** | **P-Heterogeneity** |
| --- | --- | --- | --- | --- | --- | --- |
| Executive Function |  |  |  |  |  |  |
|  | MCI | 4 | 0.16 (0.07, 0.26) | < 0.001 | 0.00% | > 0.05 |
|  | Healthy | 2 | 0.49 (0.01, 0.96) | 0.046 | 85.02% | 0.01 |
| Global Cognitive Function |  |  |  |  |  |  |
|  | MCI | 6 | 0.54 (0.31, 0.78) | < 0.001 | 66.63% | 0.01 |
|  | Healthy | 4 | 0.41 (0.22, 0.60) | < 0.001 | 61.49% | > 0.05 |
|  | Mixed | 1 | 0.24 (0.15, 0.33) | < 0.001 | NA% |  |

**Figures S1-S7. Leave-one-out sensitivity analyses.**


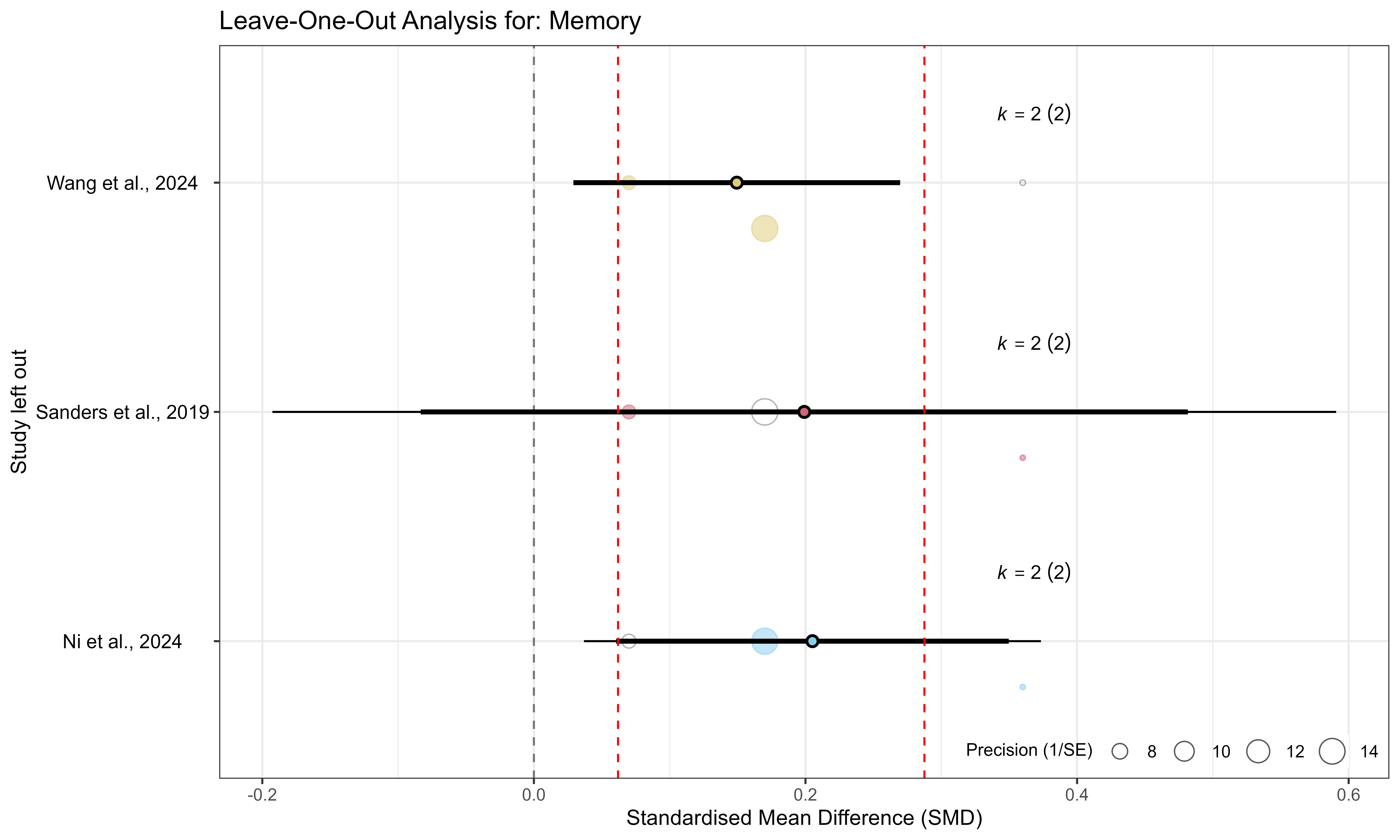

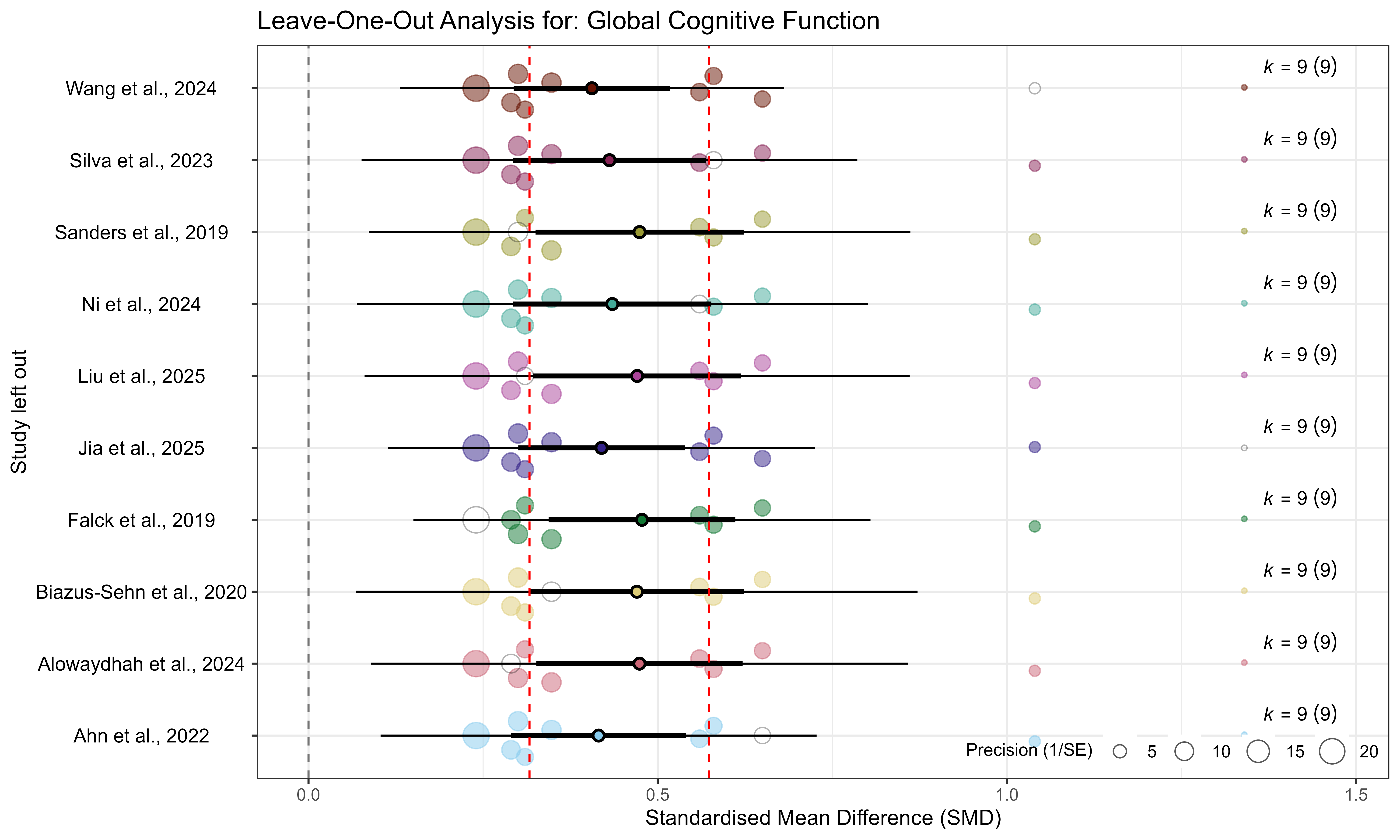

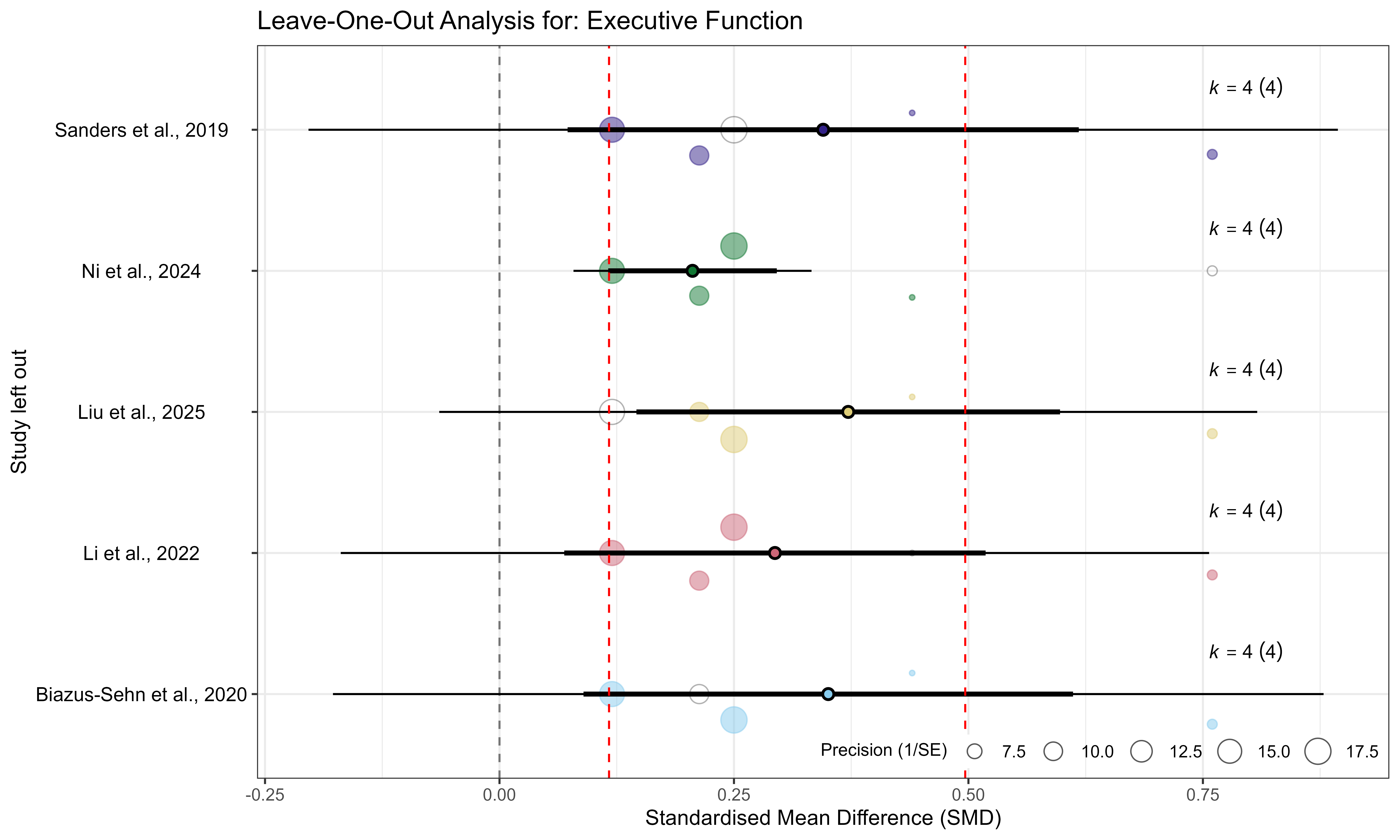

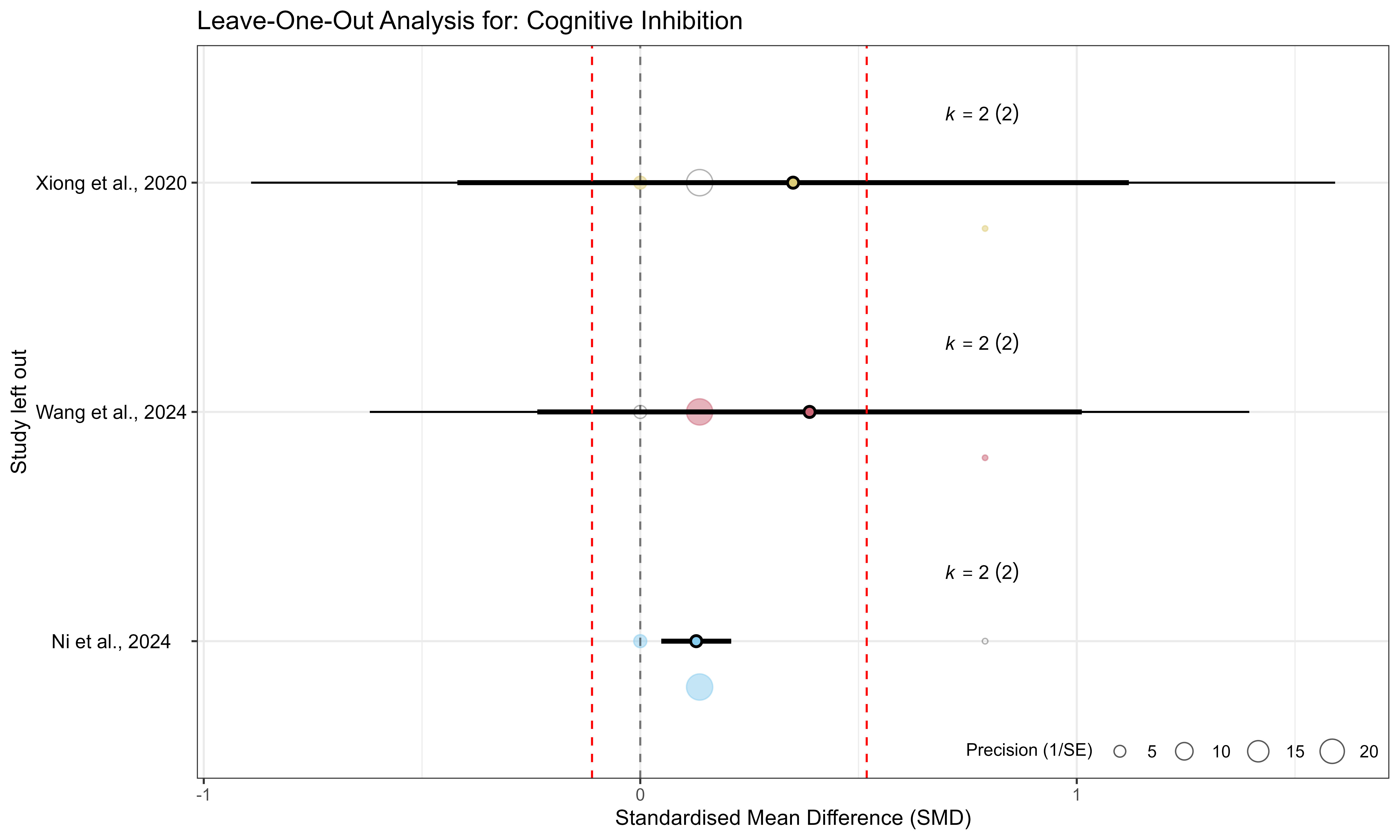

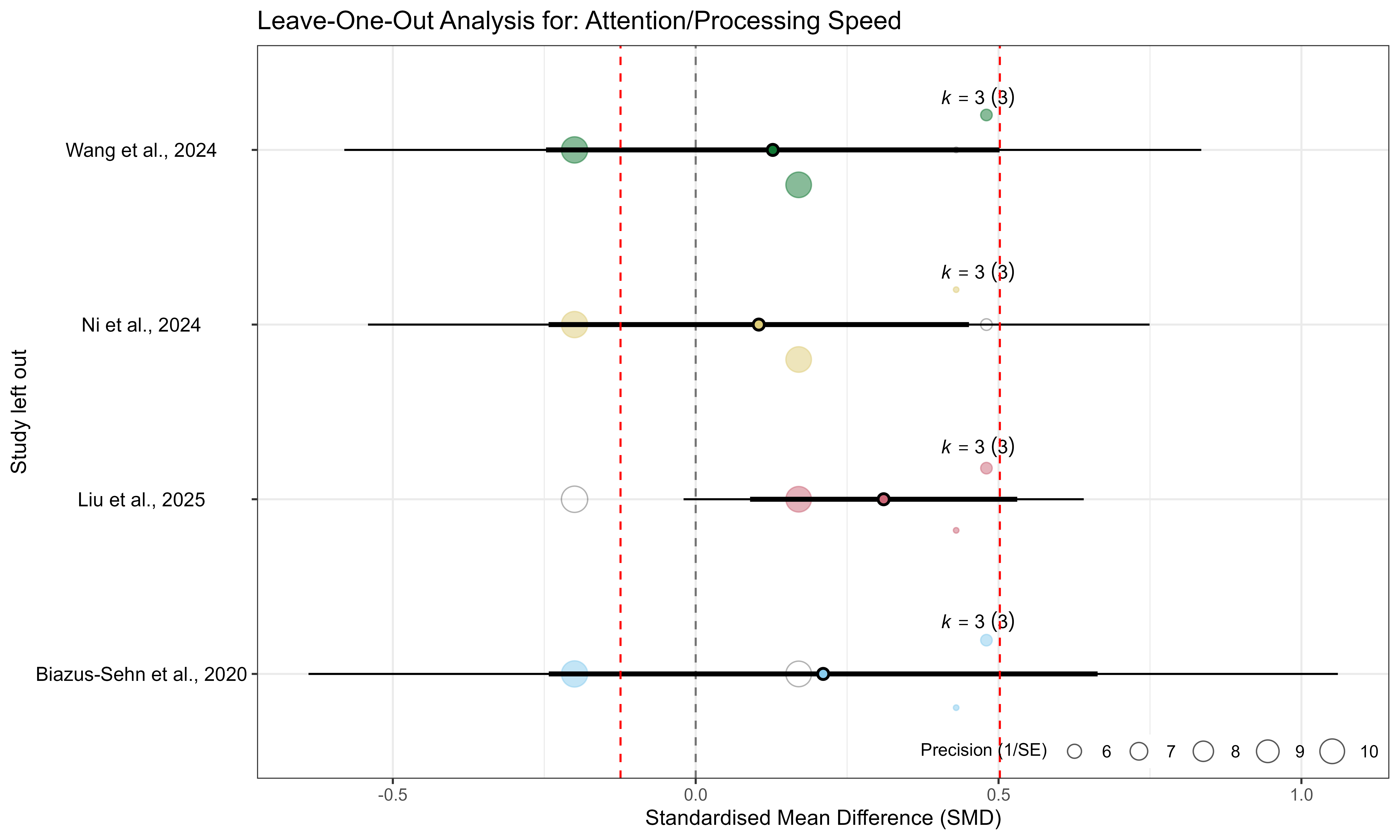

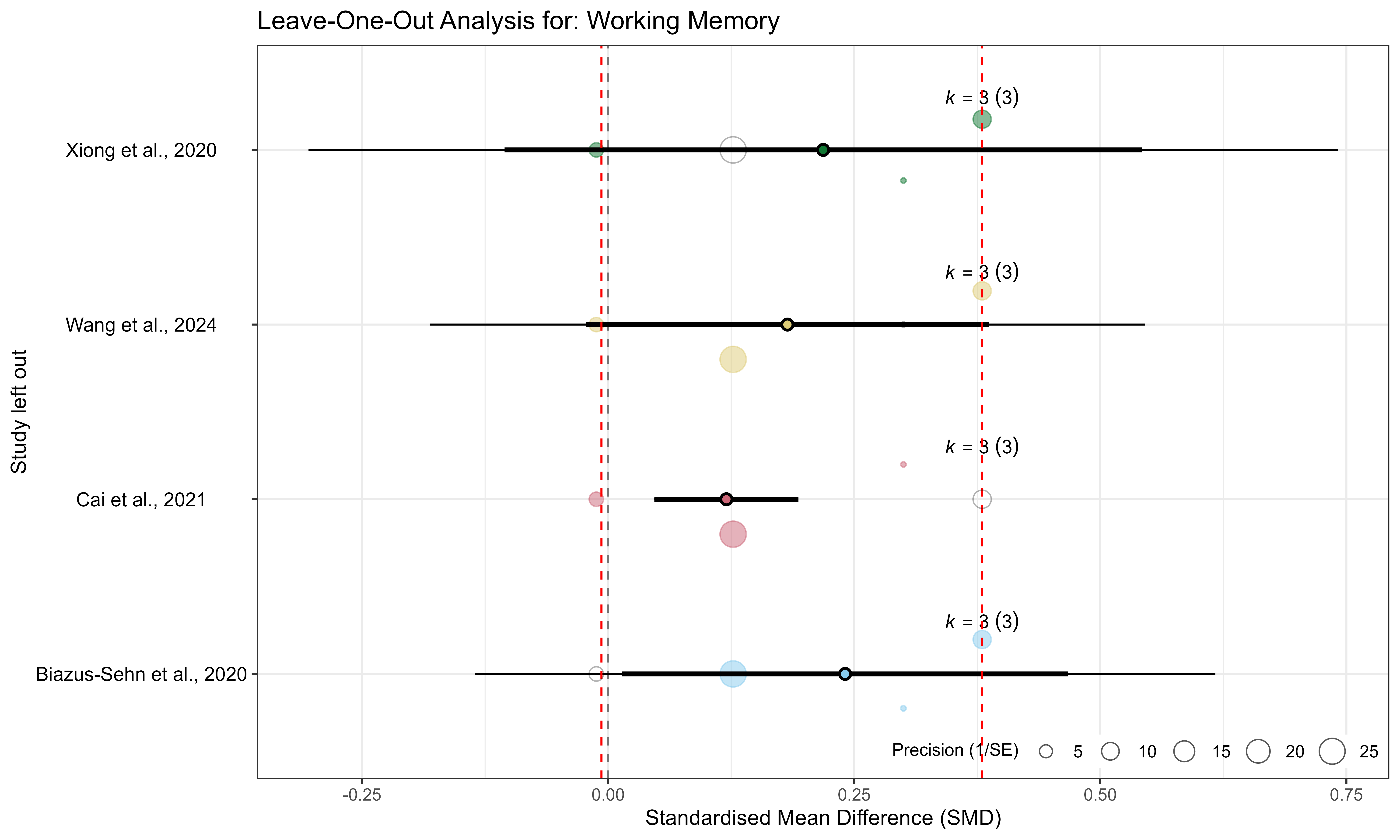

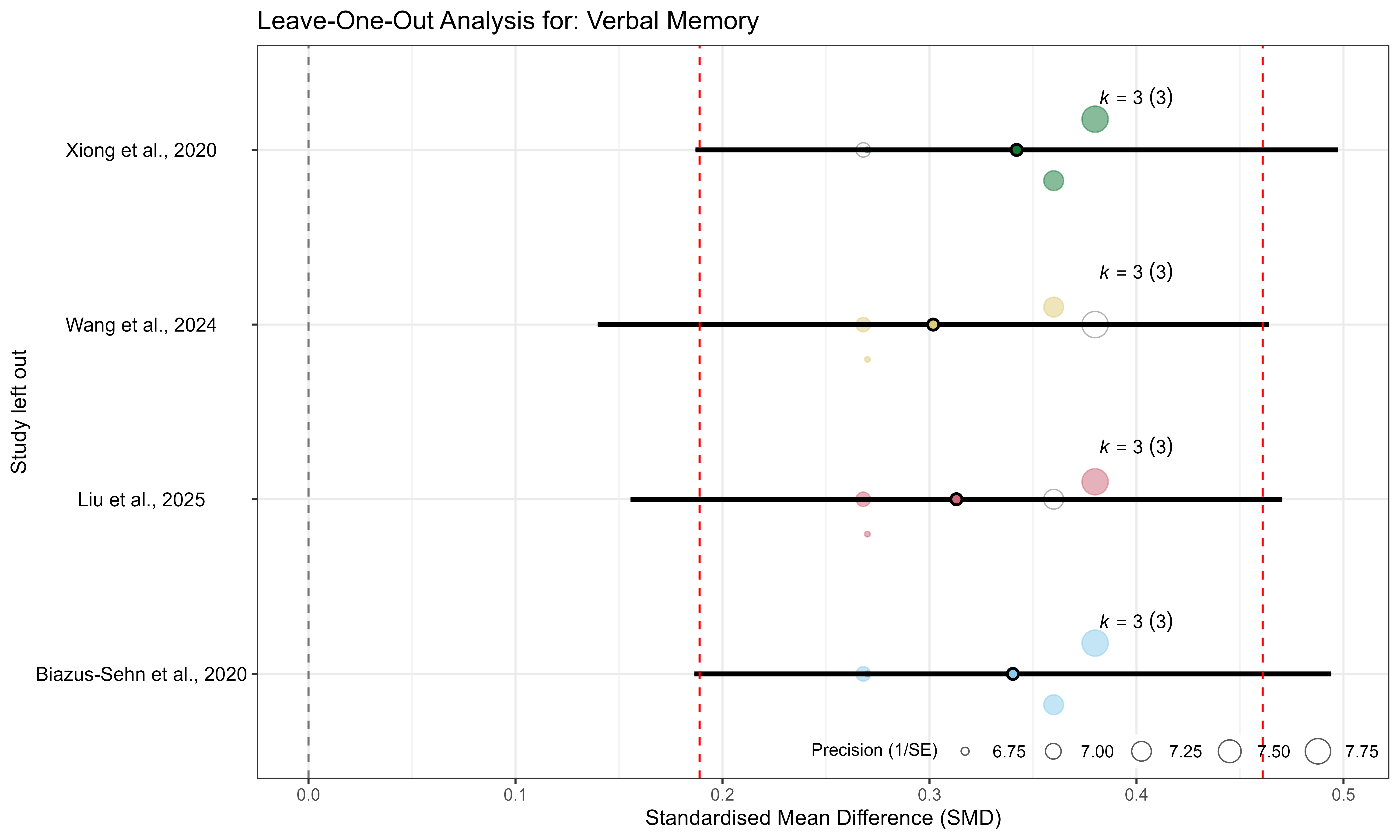

Supplement: Supplementary file 1 [file Table_1.docx]
